# PRKAR2A‐derived circular RNAs promote the malignant transformation of colitis and distinguish patients with colitis‐associated colorectal cancer

**DOI:** 10.1002/ctm2.683

**Published:** 2022-02-20

**Authors:** Daiwei Wan, Sentai Wang, Zhihua Xu, Xinquan Zan, Fei Liu, Ye Han, Min Jiang, Airong Wu, Qiaoming Zhi

**Affiliations:** ^1^ Department of General Surgery The First Affiliated Hospital of Soochow University Suzhou China; ^2^ Department of Gastroenterology The First Affiliated Hospital of Soochow University Suzhou China; ^3^ Department of Oncology The First Affiliated Hospital of Soochow University Suzhou China

**Keywords:** circular RNA, colitis‐associated colorectal cancer, PRKAR2A, RNA sequencing, *K*‐means clustering algorithm

## Abstract

**Background:**

Emerging studies have proved that colonic inflammation caused by refractory inflammatory bowel disease (IBD) can initiate the colitis‐associated cancer (CAC), but the transition from inflammation to carcinoma is still largely unknown.

**Methods:**

In this study, mouse colitis and CAC models were established, and the RNA‐seq by circRNA microarray was employed to identify the differentially expressed circRNAs and mRNAs in different comparisons (DSS vs. NC and AOM/DSS vs. DSS). The bioinformatics analyses were used to search the common characteristics in mouse colitis and CAC.

**Results:**

The *K*‐means clustering algorithm packaged these differential expressed circRNAs into subgroup analysis, and the data strongly implied that mmu_circ_0001109 closely correlated to the pro‐inflammatory signals, while mmu_circ_0001845 was significantly associated with the Wnt signalling pathway. Our subsequent data in vivo and in vitro confirmed that mmu_circ_0001109 could exacerbate the colitis by up‐regulating the Jak‐STAT3 and NF‐kappa B signalling pathways, and mmu_circ_0001845 promoted the CAC transformation through the Wnt signalling pathway. By RNA blasting between mice and humans, the human RTEL1‐ and PRKAR2A‐derived circRNAs, which might be considered as homeotic circRNAs of mmu_circ_0001109 and mmu_circ_0001845, respectively, were identified. The clinical data revealed that RTEL1‐derived circRNAs had no clinical significance in human IBD and CAC. However, three PRKAR2A‐derived circRNAs, which had the high RNA similarities to mmu_circ_0001845, were remarkably up‐regulated in CAC tissue samples and promoted the transition from colitis to CAC.

**Conclusions:**

Our results suggested that these human PRKAR2A‐derived circRNAs could be novel candidates for distinguishing CAC patients and predicted the prognosis of CAC.

## Graphical abstract

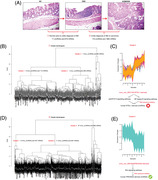

## 1 INTRODUCTION

The causal relationships between inflammation and cancer are now widely recognized and discussed. Experimental or epidemiological reports have documented that individuals with inflammatory bowel disease (IBD), for instance ulcerative colitis (UC) and Crohn's disease (CD), are more likely to develop colorectal cancer (CRC) than the general population.^1^, ^2^, ^3^ According to the large meta‐analysis in 2001 by Eaden et al., the risk of cancer progression in patients with IBD is time dependent, which may escalate 2% by 10 years, 8% by 20 years and up to 18% by 30 years.^4^ Presently, the incidence of IBD‐associated CRC declines compared with prior decades because of more safer and effective therapies to control inflammation and more improved colonoscopic technologies.^5^, ^6^ A recent meta‐analysis from Bye et al. reported that colonoscopy surveillance resulted in a significant decline in IBD‐linked CRC initiation by 42%, in comparison with the patients who did not undergo surveillance.^7^ Nevertheless, colitis‐associated cancer (CAC) still causes a significant proportion of IBD‐related deaths.^8^, ^9^ Numerous studies have showed that chronic intestinal inflammation produces pro‐inflammatory mediator production and cell proliferation, affects immune systems, and promotes carcinogenesis in CAC. Growing evidence also proves that CAC is sustained and promoted by inflammatory signals from the surrounding microenvironment.^10^, ^11^ For example, it was reported that the proinflammatory mediators or signals, such as iNOS/NO system^12^ and TLR4/NF‐κB signalling in TNF‐α/iNOS induction,^13^ provided a mechanistic relationship between inflammation and cancer. Though these mechanisms by which chronic inflammation initiates colonic carcinogenesis are being investigated, many unanswered questions remain.^14^

CircRNA (Circular RNA) is a newly discovered kind of non‐coding RNAs, which is distinguished by a covalently closed structure without 5′‐3′ polarity or a polyadenylated tail. These features make circRNA relatively stable, conserved as well as resistant to exonucleolytic RNA degradation, in comparison with its corresponding source mRNA.^15^, ^16^ CircRNAs exhibit cell‐type‐distinct and developmental‐stage‐distinct expression, which may also indicate their potential participation in diverse pathophysiological or physiological processes.^16^, ^17^, ^18^ Recent investigations have revealed that circRNAs are implicated in the CRC progression by diverse mechanisms, of which interrelating with RNA binding proteins (RBPs) and acting as miRNA sponges to control the target gene expression are the two most important ones.^19^, ^20^, ^21^ Hsa_circ_101555, for instance, had been highlighted as a novel prognostic indicator in CRC patients due to its involvement in the initiation of CRC.^21^ Also, in our study, hsa_circ_102049 was predicted to be differentially expressed during the pathology of colorectal liver metastasis. Our results showed that hsa_circ_102049 significantly promoted CRC metastasis through the circ102049‐miR‐761/miR‐192‐3p‐FRAS1 cascade. Notably, hsa_circ_102049 could also recruit and distribute DGCR8 protein to reduce the levels of mature miRNAs in the cytoplasm indirectly.^22^ However, the functions and clinical applications of circRNAs in IBD or CAC have not been reported.

In our current study, we employed the widely used dextran sodium sulphate (DSS)‐triggered acute colitis and azoxymethane (AOM)/DSS‐induced CAC models to screen the circRNA and mRNA expression profiles in inflammation and inflammation‐associated cancer by RNA‐seq. Then the differentially expressed circRNAs, mRNAs and changes of pathways during the mouse colitis and colitis‐to‐carcinoma transition were identified. Using the *K‐*means clustering algorithm, two novel circRNAs, for example, mmu_circ_0001109 and mmu_circ_0001845, were proved to associate with the colitis and transition of colitis to CAC, respectively. In addition, we blasted the regulator of telomere elongation helicase 1 (RTEL1)‐ or RII alpha (Type II alpha regulatory subunit) of cAMP‐dependent protein kinase (PRKAR2A)‐derived circRNAs in humans, and evaluated their expressions in different sample comparisons (healthy controls, UC and CAC patients). Comparing the UC onset to surgery and overall survival (OS) rates according to the expressing levels of circular RNAs, three PRKAR2A‐derived circRNAs in humans were finally proved to couple to oncogenic characteristics, and could be used as clinical candidate markers of human CAC.

## 2 MATERIALS AND METHODS

### 2.1 Cell culture and samples

Murine colon carcinoma cells (CT‐26 and MC‐38) and human CRC cells (HCT116, DLD‐1, HT‐29, SW1116, KM12, SW408 and LoVo) were acquired from the ATCC (American Type Culture Collection, Manassas, VA, USA), and inoculated in DMEM (Thermo, USA) or RPMI‐1640 (Invitrogen, USA) enriched with FBS (10%), streptomycin and penicillin at 37°C and 5% CO_2_. Clinical samples were collected from 56 UC, 72 CAC patients and 86 healthy controls, who underwent surgery or biopsy from 2008 to 2015 at The First Affiliated Hospital of Soochow University. Patients with CAC were diagnosed with colorectal adenocarcinoma in the chronic inflammatory lesions of underlying UC during follow‐up periods, and received surgical excisions. The specimens were snap‐frozen in liquid nitrogen after biopsy or surgical excision immediately. The demographic and clinical characteristics of all these UC and CAC patients are presented in Table S1. This research work was granted approval by the ethics committee of The First Affiliated Hospital of Soochow University.

### 2.2 Animals and experimental groups

Thirty 6‐week‐old male mice (BALB/c, 18–20 g in weight) were classified into three groups: the blank control (NC, *n* = 10), colitis (DSS, *n* = 10) and CAC group (AOM/DSS, *n* = 10). Mice of the NC group drank water for 75 days. Colitis was induced by replacing drinking water with 3.0% DSS (35 000–50 000, MW, MP Biomedicals, USA) for 7 days. For the mouse CAC model, we intraperitoneally inoculated mice with AOM (12.5 mg/kg). After 5 days, DSS (3.0%) was added into the drinking water for 7 days, and subsequent 14 days of regular water. We repeated this cycle by three times. When mice were sacrificed, the colons were collected from the ileocecal junction to anus, and opened longitudinally. All experimental procedures were granted approval by the Experimental Animal Care and Use Committee (The First Affiliated Hospital of Soochow University).

1 **HIGHLIGHT**

- RNA sequencing was performed to screen the differentially expressed circRNAs and mRNAs at the colitis and CAC stages.
- *K*‐means clustering algorithm implied that mmu_circ_00001109 (or mmu_circ_0001845) might play a promoting role during the inflammation (or colitis‐to‐carcinoma transition).
- RNA sequence blasting suggested that the PRKAR2A‐derived circRNAs promoted the malignant transformation of colitis and distinguish patients with CAC.

### 2.3 Haematoxylin and eosin and alcian blue staining

The fixed colon tissues (10% neutral buffered formalin [NBF]) were processed via graded alcohol, xylene, as well as paraffin, and finally paraffin‐blocked. Segments (4 μm thickness) were prepared, and followed by haematoxylin and eosin (H&E) staining. Then H&E‐stained sections were observed, and the histological score according to the crypt damage, infiltration of neutrophils, and foci of ulceration in the detected colons was observed and calculated.^23^ Goblets (stained blue with 1% alcian blue 8GX) were counted per colon tissue filed (20×). Two pathologists blind to this study completed the histological examinations.

### 2.4 RNA‐Seq by circular RNA microarray

The Agilent‐085631 was utilized in our study, and the OE Biotech (Shanghai, China) performed the RNA‐Seq data analysis (15 samples). Isolation of total RNA from the mice colons was done with the TRIzol reagent (Life Tech, USA). Afterwards, the NanoDrop 2000/2000c (Thermo Fisher Scientifc, USA) and Agilent Bioanalyzer 2100 (Agilent Tech, USA) were employed to assess the quantity and the quality of the RNA, respectively. After that, the labelling of samples, microarray hybridization along with washing were conducted by the manufacturer. Double strand cDNA was generated from the RNA, converted into cRNA, and subsequently conjugated to cyanine‐3‐CTP. Then, hybridization of the conjugated cRNAs onto the microarray was done. After washing, the Agilent Scanner G2505C (Agilent Tech, USA) was used to screen the arrays. The raw data were generated from array images by the Feature extraction software (V10.7.1.1, Agilent Tech, USA). We employed Genespring (V14.8, Agilent Tech, USA) to complete the basic analysis from the raw data. Briefly, normalization of the raw data was done with the quantile algorithm. After that, we selected the probes exhibiting at least one condition out of two conditions with flags in ‘Detected’ for subsequent data analysis. Differentially expressed circRNAs or genes were then determined via fold change along with *p*‐value, which was computed with *t*‐test. The OE Biotech's software was used to verify the circRNAs. ‘Mapped backsplicing junction reads per million mapped reads’ (RPM) as described previously^24^ was employed to detect the levels of circRNAs. All circRNAs had been annotated with the Circbase website (http://www.circbase.org/). The data discussed in this publication had been uploaded to the website of NCBI (https://www.ncbi.nlm.nih.gov/geo/query/acc.cgi?acc = GSE166708).

### 2.5 Detection of differentially expressed circRNAs and genes

Differentially expressed circRNAs and genes were determined via fold change along with *p*‐value computed through *t*‐test. A fold change of ≥2.0 along with a *p* ≤.05 were served as the cut‐off for up‐ or down‐regulated molecules. CircRNAs or mRNAs were filtered out in pairwise groups. The differentially expressed circRNAs and mRNAs were depicted in squares with distinct colours. Hierarchical clustering was performed to describe the expression patterns of differentially expressed circRNAs or mRNAs among different samples.

### 2.6 The gene ontology/Kyoto encyclopaedia of genes and genomes analysis

The GO assessment was done to determine biological implications of unique genes in the remarkable or representative patterns of the genes that are differentially expressed (or parental genes of circRNAs). The GO categories, which originated from the Gene Ontology website (http://www.geneontology.org), constituted three structured networks consisting of defined terms describing gene product attributes. According to the KEGG data (https://www.kegg.jp/), cascades analyses were adopted to assess the significant pathways from the differentially related mRNAs.

### 2.7 *K*‐means clustering algorithm

Through the implementation of *K‐*means clustering algorithm in R (*K‐*means package) as described previously,^25^, ^26^, ^27^ the clustering was conducted. By the *K‐*means clustering, genes with similar expressing patterns were defined and divided as gene modules. The circRNAs and genes within the same group (module) were of similar expression patterns, and might have common functions. Based on such inter‐category similarity, *K*‐means = 3 was selected as the optimal one.

### 2.8 Co‐expression network construction

According to the expressions of significantly dys‐regulated circRNAs and protein‐coding mRNAs, the co‐expression analysis was performed by Pearson's correlation coefficient. A coefficient parameter value of >0.6 (or ← 0.6) with *p* < 0.05 was signified as statistical significance.

### 2.9 Fluorescence in situ hybridization

According to the instructions, the fluorescence in situ hybridization (FISH) analysis was performed by the FISH Kit (F21201, Genepharma, Shanghai, China). Cy3‐labeled mmu_circ_0001109 (5′‐CACGCUGAACACAAUCUUGUUUCCUGGAUGCAGU‐3′) or mmu_circ_0001845 (5′AUUUGCUAGGAACCGGAACAAUCCCCAAAGGGAGC‐3′) probes were also designed and obtained from Shanghai GenePharma (China). After the slides were dewaxed, the probes were added into the hybridization solution and incubated with cells overnight in the dark at 37°C. Subsequently, slides were incubated with the following primary phospho‐STAT3 (1: 200, YP0250, Immunoway, USA), phospho‐P65 (1: 400, YP0847, Immunoway, USA) and β‐catenin (1: 400, #8814, CST, USA) for 2 h at 37°C. Then, slides were incubated with secondary antibody (BS50950, 1: 100, Bioworld, USA). Finally, the slides were counter stained with DAPI and imaged. The fluorescence intensity was calculated by the Image J software.

### 2.10 Quantitative real‐time polymerase chain reaction

Total RNA was isolated was from mouse tissue samples or cell lines using the TRIzol regent (Invitrogen, USA). The Prime Script RT Master Mix (Takara, Japan) was purchased to synthesize the cDNA, and the FastStart Universal SYBR Green Master (Roche, Germany) was used for the quantitative real‐time polymerase chain reaction (qRT‐PCR) analysis. Finally, the data was analysed with the LightCycler 96 System (Roche, Germany), and GAPDH was considered as the internal control. The primers of this study were presented in Table S2.

### 2.11 Plasmids construction and cell transfection

We synthesized the circRNA (mmu_circ_0001109 or mmu_circ_1845) cDNA, and subsequently cloned them into the pCD5‐ciR vector (Geneseed, Guangzhou, China). OE‐NC (transfected with over‐expression NC vector), OE‐mmu_circ_0001109 (transfected with mmu_circ_0001109 over‐expression vector) or OE‐mmu_circ_0001845 (transfected with mmu_circ_0001845 over‐expression vector) vectors were transfected with the Lipofectamine 3000 agent. After transfection at 48 h, the efficiency of cell transfection for each group was evaluated by PCR. Similarly, the hsa_circRNA (hsa_circ_0124022, hsa_circ_0124028 or hsa_circ_0124029) cDNA was also synthesized, which was subsequently cloned into the pCD5‐ciR vector correspondingly.

### 2.12 Western blot

Tissue or cellular protein was extracted using the RIPA lysis buffer (Beyotime, Shanghai, China), and the cytoplasmic and nuclear fractions were isolated by the extraction kit (P0027, Beyotime, Shanghai, China). The protein quantification was done with the BCA protein assay kit (Thermo Fisher Scientific, USA). The protein extracts were isolated with SDS‐PAGE gel (12%), which were subsequently transferred to the nitrocellulose membranes. The membranes were blocked for 1 h and incubated with following primary STAT3 (1:2000, 60199‐1‐Ig, Proteintech, USA), phospho‐STAT3 (1:1000, YP0250, Immunoway, USA), P65 (1:1000, 66535‐1‐Ig, Proteintech, USA), phospho‐P65 (1:1000, YP0847, Immunoway, USA), IκBα (1:1000, YM3718, Immunoway, USA), β‐catenin (1:1000, #8814, CST, USA), phospho‐β‐Catenin (1:1000, #4176, CST, USA), GSK3β (1: 5000, ab32391, Abcam, USA), Cyclin D1 (1:10000, ab134175, Abcam, USA), LEF‐1 (1:1000, ab131872, Abcam, USA), TCF‐1 (1:1000, #2203, CST, USA) antibodies overnight at 4°C and HRP‐labelled secondary antibodies (1:4000, Sigma, USA) for 2 h at room temperature. The ECL kit (Thermo Fisher Scientific, USA) was utilized to determine the immunoreactive bands, and the band densities were normalized to β‐actin (1:3000, cw0096, CWbiotech, China).

### 2.13 Luciferase reporter assay

Cells were planted into the 24‐well plate (5 × 10^4^) and allowed to settle for 12 h. The luciferase reporter analysis was performed using the TOP along with FOP reporters (Upstate Biotech), which consisted of the wild‐ and mutated‐type of TCF/LEF DNA binding sites. Using the Lipofectamine 3000 agent (Invitrogen), the pTK‐RL and TOP‐Flash reporter plasmids were co‐transfected transiently into the corresponding cells. After transfection for 4 h, the luciferase reporter assay system (Promega, USA) was employed to determine the activities of firefly and Renilla luciferase reporters.

### 2.14 Statistical analyses

Statistical analyses were implemented in the software of SPSS 19.0 (SPSS Inc., Chicago, USA). The data was described as mean ± standard deviation from experiments in replicate. Statistically differences were estimated by one‐way ANOVA, which was followed by the Tukey test. The relationships between circRNAs (mmu_circ_0001109 or mmu_circ_0001845) and signalling proteins (p‐STAT3, p‐P65 or β‐catenin) were assessed by the spearman Pearson correlation analysis. The survival curves were plotted by the Kaplan‐Meier approach using the log rank test. The relationships between human PRKAR2A‐derived circRNAs (hsa_circ_0124022, hsa_circ_0124028 and hsa_circ_0124029) and clinicopathological characteristics of 72 CAC patients were calculated by Pearson χ2 test and Fisher's exact test as per data type. *p* < 0.05 signified statistical significance.

## 3 RESULTS

### 3.1 Identifications of circRNAs in the mouse colitis and CAC models

**FIGURE 1:**
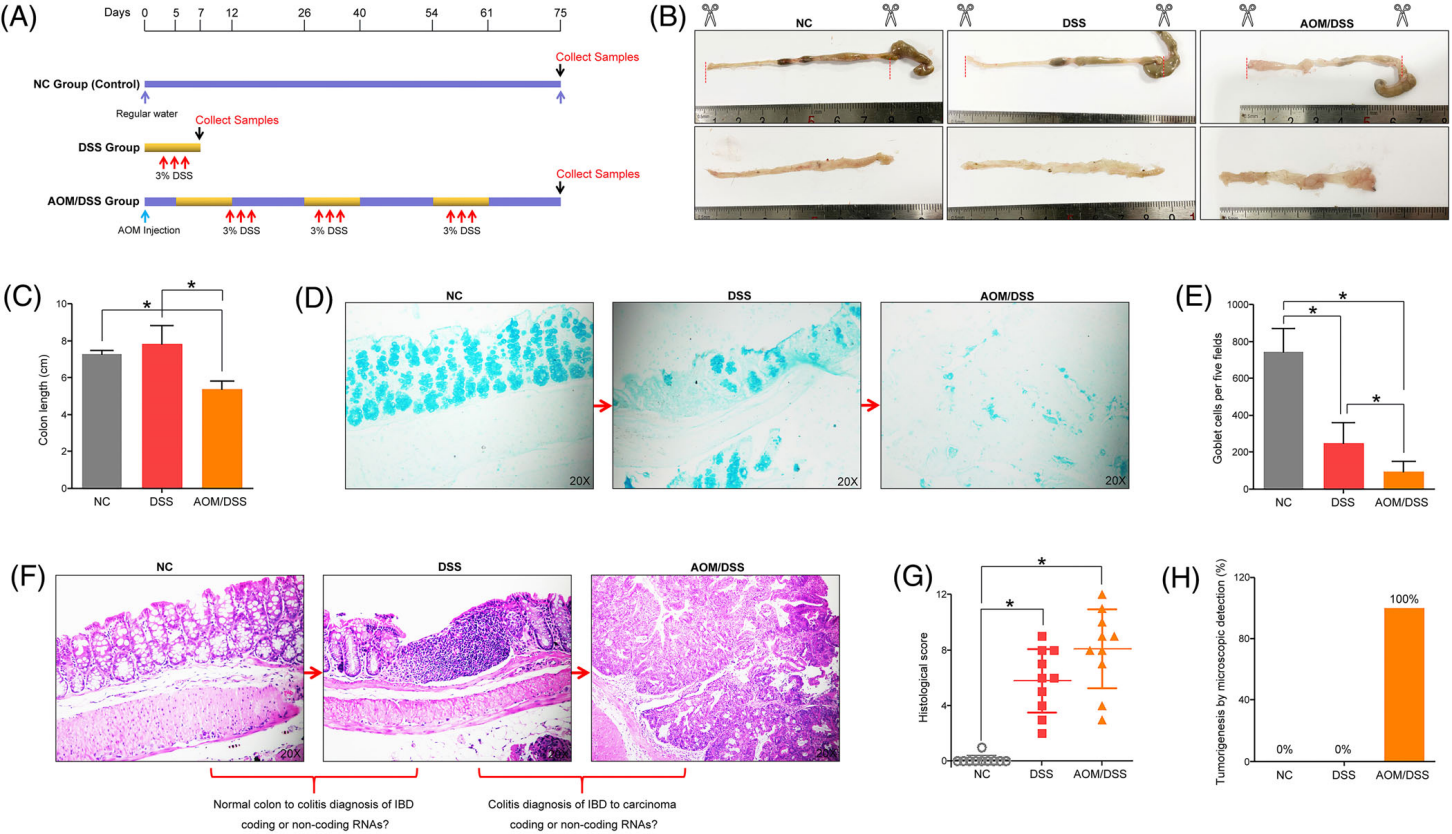
Establishment of mouse colitis and colitis‐associated cancer (CAC) models by DSS or AOM/DSS treatment. (A) The scheme for colitis and CAC induction. (B and C) Upon sacrifice, the colons were excised from the ileocecal junction to the anus, and the colon lengths of mice were calculated. (D and E) Goblets were stained blue with 1% alcian blue and counted per colon tissue filed. (F and G) Haematoxylin and eosin (H&E) staining was used to calculate the histological score of mice colons. (H) The percentages of tumourigenesis were observed under the microscope (*p < .05)

**FIGURE 2:**
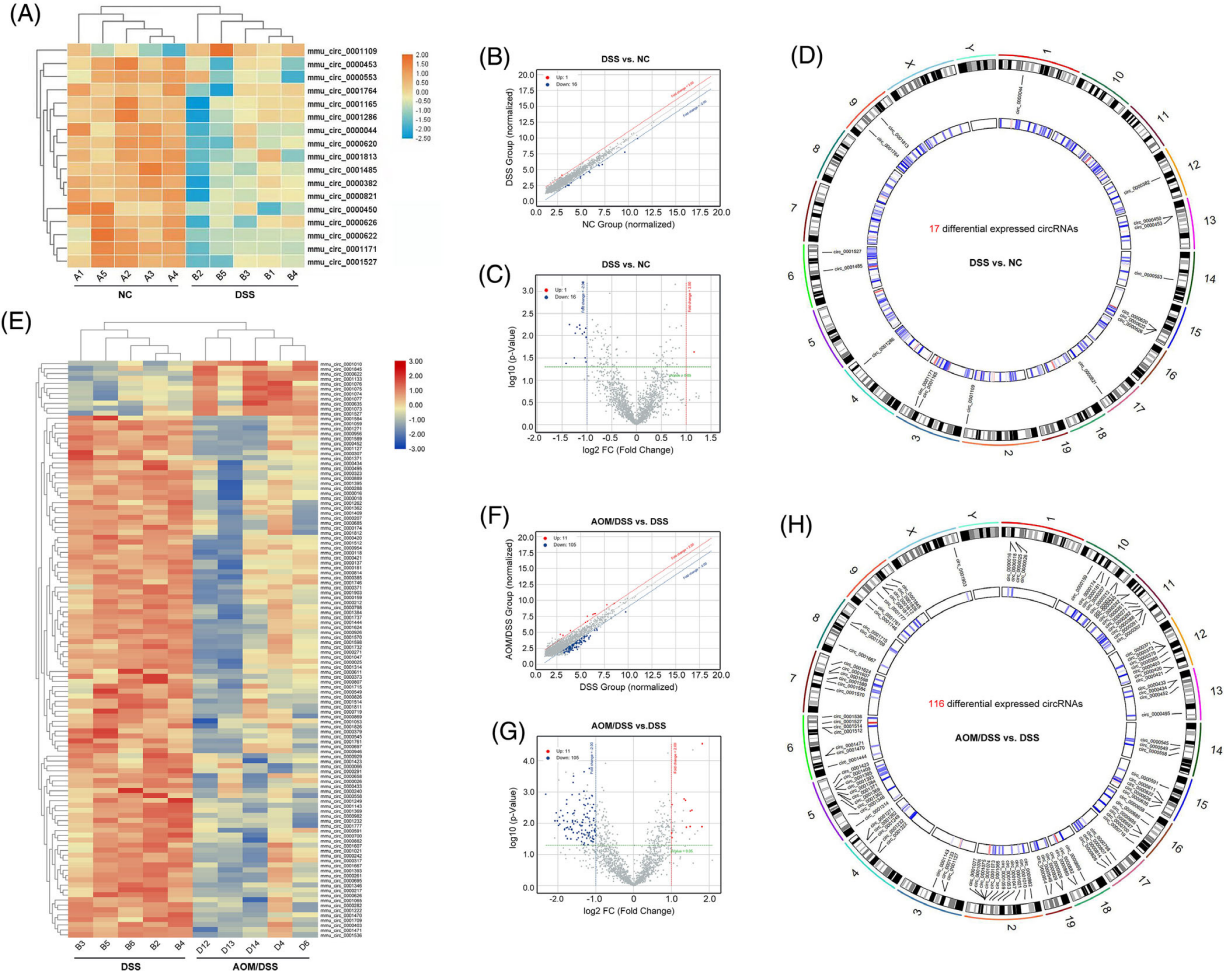
The differentially expressed circRNAs and mRNAs were identified by the throughput RNA sequencing. (A–D) The hierarchical clustering heat map, volcano and scatter plot showed 17 differentially expressed circRNAs in the colitis (DSS vs. NC). The circos plot described the positions of 17 circRNAs on mouse genome assembly mm9. (E–H) The hierarchical clustering heat map, volcano and scatter plot showed 116 differentially expressed circRNAs during the inflammation‐to‐carcinoma transition (AOM/DSS vs. DSS). The circos plot described the positions of 116 circRNAs on mouse genome assembly mm9

In order to perform the RNA sequencing, at first, we successfully established the mouse colitis and CAC models as described in Figure 1A. The colon length of CAC mice was significantly shortened, compared to that of the normal or DSS‐induced colitis, and most of the carcinomas were located in the mid or distal region of the colon, with only occasional ones observed in the proximal colon (Figure 1B,C). Histological examination revealed that oral DSS administration for 7 days induced goblet cell and crypt loss, and severe inflammatory infiltrate in C57BL/6 mice, while AOM/DSS treatment led to submucosal carcinomas with chronic inflammations and/or regenerative changes (Figure 1D–G). The histopathological observations under microscope also confirmed the repeatability of mouse colitis and CAC models for subsequent RNA sequencing (Figure 1H). To reveal the disease‐related progressions, we compared the microarray data, and screened the significant circRNAs in each two‐state comparison (DSS vs. NC and AOM/DSS vs. DSS). As shown Figure 2A–C, the results of hierarchical clustering heat map, scatter plot and volcano plot demonstrated that 17 differentially expressed circRNAs were included in the DSS‐induced colitis, and these 17 circRNAs were distributed on diverse chromosomes widely (Figure 2D; Table S3). The number of differentially expressed circRNAs between AOM/DSS and DSS group was greater, and a total of 116 circRNAs were involved during the inflammation‐to‐carcinoma transition (Figure 2E–H, Table S4).

### 3.2 The GO and KEGG enrichment in the mouse colitis and CAC models

**FIGURE 3:**
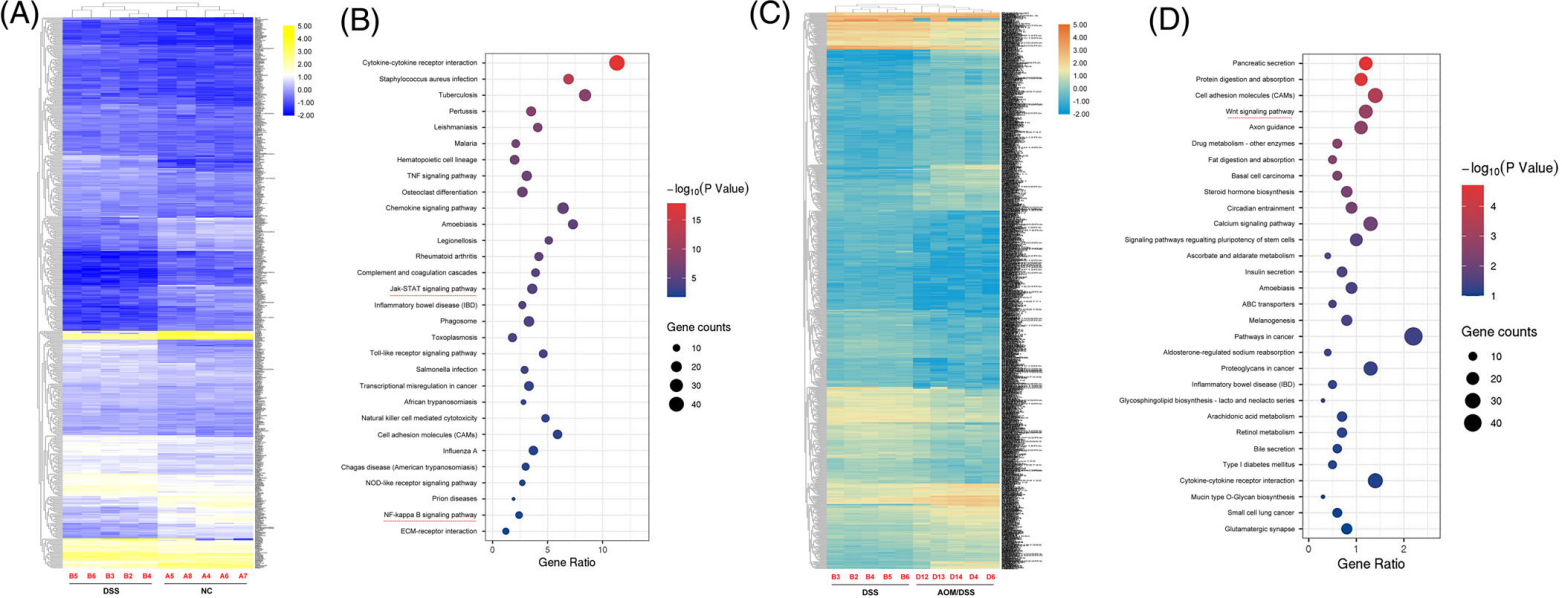
The KEGG enrichment in the mouse colitis and colitis‐associated cancer (CAC) models. (A and B) The KEGG analysis from differentially expressed mRNAs (DSS vs. NC) showed the top 30 enriched signalling pathways in the colitis. (C and D) The KEGG analysis from differentially expressed mRNAs (AOM/DSS vs. DSS) showed the top 30 enriched signalling pathways during the inflammation‐to‐carcinoma transition

Besides, the global expression profiles of mRNAs in each two‐state comparison were also determined, and 679 differentially expressed mRNAs were identified and changed between the DSS and NC groups (Figure 3A). As our previous reports,^15^ the Gene Ontology/Kyoto Encyclopaedia of Genes and Genomes (GO/KEGG) analysis based on the parental genes of circRNAs might be used to predict the most enriched terms, including molecular functions, cellular components, biological processes and signalling pathways. But in our study, the bioinformatics analyses from the parental genes in each state comparison did not show any useful information for us. The enriched GO or pathways had no significant connections with inflammation or carcinoma (Figure S1A–D). Thus, the GO biological process classification from differentially expressed mRNAs was calculated, and the top 10 biological processes, such as cellular response to interleukin‐1, innate immune response, cellular response to interferon‐beta, neutrophil chemotaxis, chemotaxis, immune response, cellular response to interferon‐gamma, immune system process and inflammatory response were included during the DSS‐induced colitis (Figure S2A). Meanwhile, the KEGG enrichment was used to reveal the pathways, which might relate to these differentially expressed mRNAs. Our data elucidated that several inflammation‐related pathways were significantly enriched, including the chemokine signalling pathway, TNF signalling pathway, Jak‐STAT signalling pathway, IBD, Toll‐like receptor signalling pathway and NF‐kappa B signalling pathway (Figure 3B). Similarly, 1960 differentially expressed genes (AOM/DSS vs. DSS) were screened (Figure 3C), and the carcinoma‐related biological processes such as negative regulation of angiogenesis, negative regulation of Wnt signalling pathway, cell adhesion and cell‐matrix adhesion were included in the inflammation‐to‐carcinoma transition (Figure S2B). Besides, the KEGG enrichment revealed that the Wnt signalling pathway might be closely associated with the CAC transformation (Figure 3D).

### 3.3 *K*‐means clustering algorithm of differentially expressed circRNAs and mRNAs in the mouse colitis model

**FIGURE 4:**
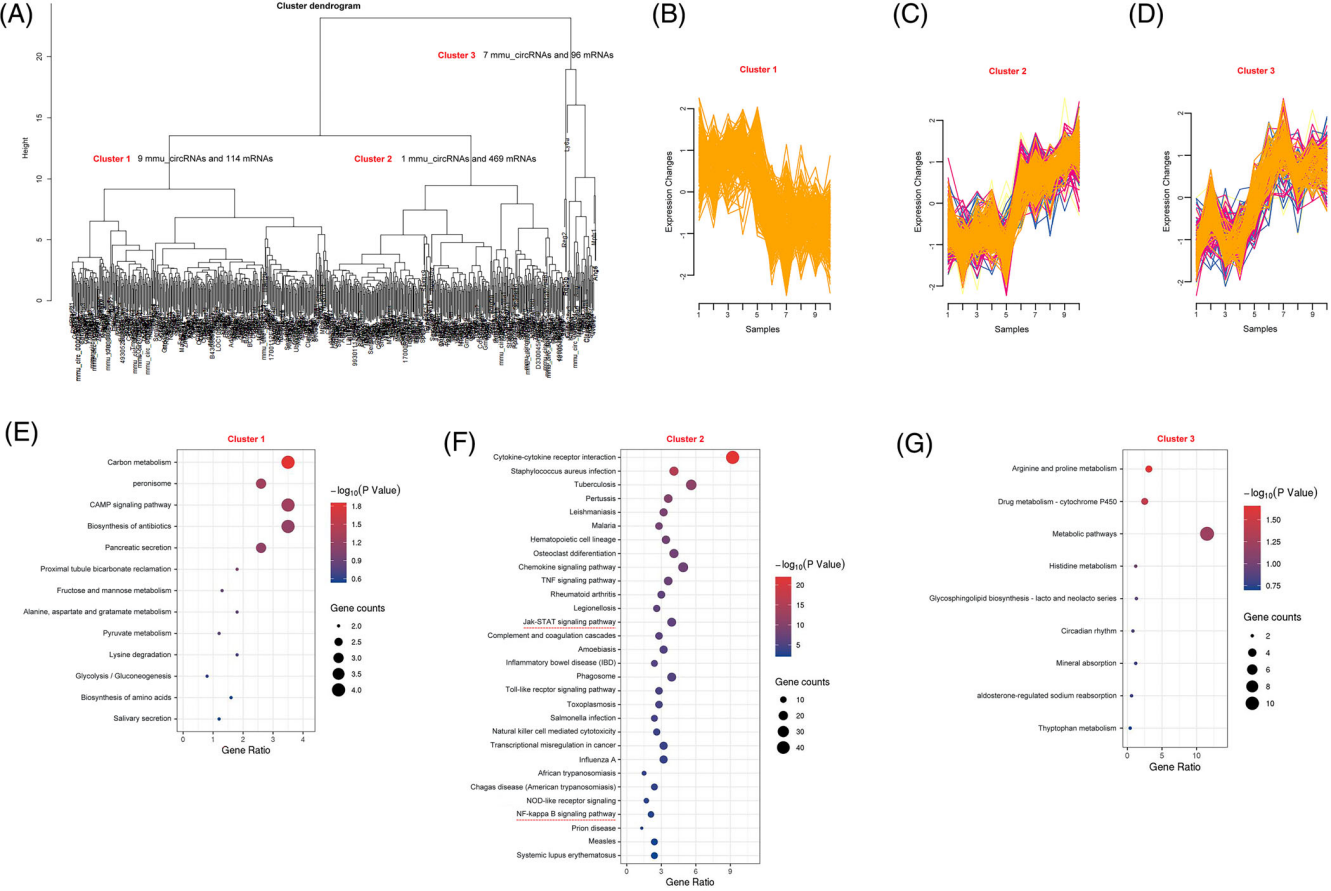
The *K*‐means clustering algorithm divided the differentially expressed circRNAs and mRNAs of colitis into three subgroups. (A) The scheme for *K*‐means clustering algorithm in the mouse colitis. (B–D) Cluster 1–3 with similar expression patterns were defined. (E–G) The KEGG analyses from differentially expressed mRNAs in clusters 1–3 were described

Using the *K*‐means clustering algorithm, all differentially expressed circRNAs and mRNAs were classified into 3 stable clusters (Figure 4A–D). To reveal the feature of each subtype, the GO and pathway analyses were employed to calculate the top GO and canonical pathways. As shown in Figure S3A–C, only cluster 2 was concordance with the results of GO enrichment from total differentially expressed mRNAs. These top 10 biological processes included the positive regulation of inflammation response, response to lipopolysaccharide, cellular response to interferon‐beta, innate immune response, neutrophil chemotaxis, chemotaxis, immune response, cellular response to interferon‐gamma, immune system process and inflammatory response. More interestingly, the KEGG analysis from cluster 2 also demonstrated that inflammation‐related pathways, such as Cytokine‐cytokine receptor interaction, Chemokine signalling pathway, TNF signalling pathway, Jak‐STAT signalling pathway, IBD, Toll‐like receptor signalling pathway and NF‐kappa B signalling pathway, were significantly enriched. Whereas these enriched signalling pathways from cluster 1 and 3 had little to do with the DSS‐induced inflammations (Figure 4E–G). These results provided us a useful clue that mmu_circ_0001109 might play an essential or key role during the mouse colitis.

### 3.4 Mmu_circ_0001109 exacerbated the colitis by activating the Jak‐STAT3 and NF‐kappa B signalling pathways

**FIGURE 5:**
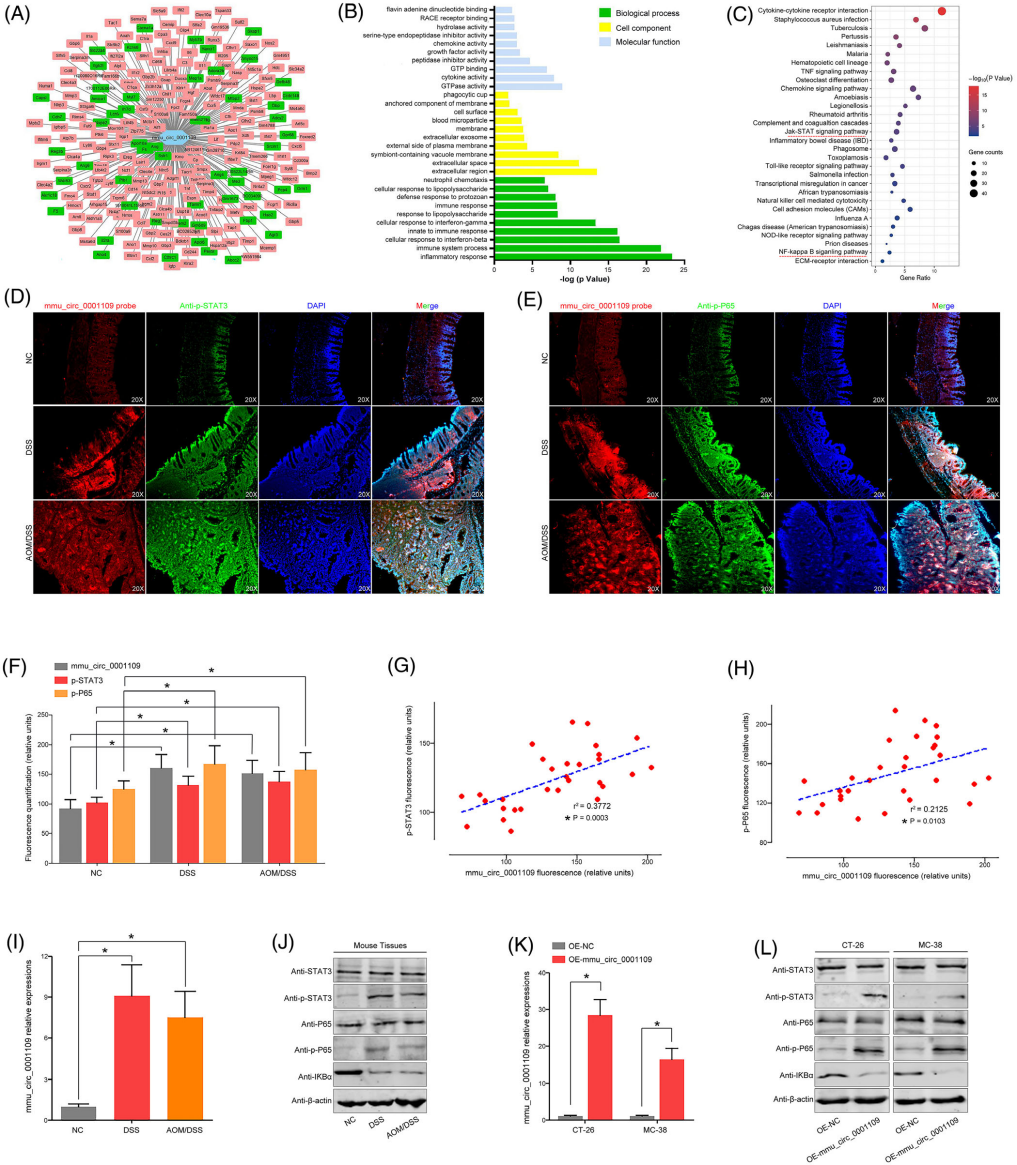
Mmu_circ_0001109 exacerbated the colitis by activating the Jak‐STAT3 signalling pathway and NF‐kappa B signalling pathway. (A) The co‐expressed genes of mmu_circ_0001109 were calculated. (B and C) The GO and KEGG analyses were done according to the mmu_circ_0001109 co‐expressed genes. (D–F) The co‐expression of mmu_circ_0001109 and p‐STAT3 (p‐P65) in mice colon was determined by immunofluorescence, and the fluorescence quantification was compared. (G and H) The Spearman–Pearson correlation between mmu_circ_0001109 and p‐STAT3 (or p‐P65) fluorescence intensity in mice was analysed. (I and J) Mmu_circ_0001109 expressions in each compared group were detected by the PCR analysis, and key proteins of the Jak/STAT3 and NF‐kappa B signalling pathways were also determined by the western bolt analysis. (K‐L) Mmu_circ_0001109 over‐expression vector was constructed and verified by the PCR analysis, which could significantly change the protein expressions of inflammation‐related signalling pathways (*p < .05)

To further reveal the potential relationships between mmu_circ_0001109 and inflammatory signalling pathways, the co‐expression analysis was performed and the network of mmu_circ_0001109‐related genes was constructed (Figure 5A). Then the GO analysis among these co‐expressed genes demonstrated that mmu_circ_0001109 significantly correlated with neutrophil chemotaxis, immune response, cellular response to interferon‐gamma, innate to immune response, cellular response to interferon‐beta, immune system process and inflammatory response (Figure 5B). Interestingly, the KEGG enrichment implied that two inflammatory signalling pathways, for example, Jak‐STAT3 and NF‐kappa B signalling pathways were also co‐related with the mmu_circ_0001109 co‐expressed genes (Figure 5C). This implied that mmu_circ_0001109 might link with the colitis through the Jak‐STAT3 or NF‐kappa B signalling pathway. Then the results of FISH assays showed that the levels of mmu_circ_001109, p‐STAT3 and p‐P65 were significantly up‐regulated in both DSS‐induced colitis and AOM/DSS‐associated CAC groups, compared to the NC group. More importantly, the tissue mmu_circ_0001109 expressions were highly positively correlated with the protein expressions of p‐STAT3 and p‐P65 (Figure 5D–H), and the subsequent experiments of PCR and western analysis confirmed our findings (Figure 5I,J). To better reveal the regulatory relationships between mmu_circ_0001109 and colitis‐related signals, we constructed the over‐expressing mmu_circ_0001109 plasmid in vitro, and the results revealed that up‐regulation of mmu_circ_0001109 in cells could simultaneously activate the Jak/STAT3 and NF‐kappa B signalling pathways in vitro (Figure 5K,L). These results implied that mouse colitis and CAC specimens had their own common characteristics and mechanisms, in which over‐expressions of mmu_circ_0001109 might significantly activate the Jak/STAT3 and NF‐kappa B signalling pathways.

### 3.5 *K*‐means clustering algorithm of differentially expressed circRNAs and mRNAs in the mouse CAC model

**FIGURE 6:**
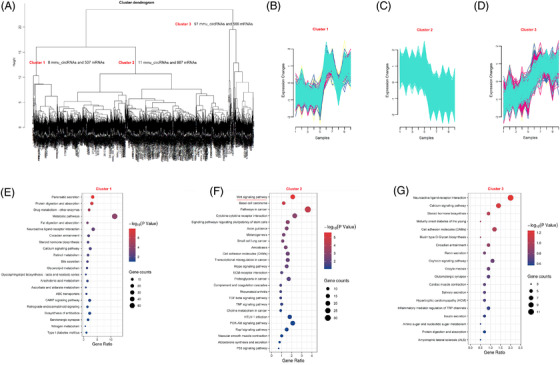
The *K*‐means clustering algorithm divided the differentially expressed circRNAs and mRNAs of inflammation‐to‐carcinoma into three subgroups. (A) The scheme for *k*‐means clustering algorithm during the mouse colitis‐associated cancer (CAC) transformation. (B–D) Cluster 1–3 with similar expression patterns were also defined. (E–G) The KEGG analyses from differentially expressed mRNAs in clusters 1–3 were described

The *K*‐means clustering algorithm was also performed to identify the useful information from colitis to CAC. As shown in Figure 6A–D, all changed circRNAs and mRNAs were divided into three subgroups. Each subgroup included the selected cirRNAs and their corresponding mRNAs. Then the GO analysis demonstrated that only cluster 2 included the cancer‐related biological processes, such as the negative regulation of cell proliferation, regulation of cell proliferation, Wnt signalling pathway, negative regulation of Wnt signalling pathway and negative regulation of canonical Wnt signalling pathway (Figure S4A–C). Similarly, the KEGG analysis in cluster 2 also enriched some cancer‐related pathways, such as the Wnt signalling pathway, Basel cell carcinoma, signalling pathways regulating pluripotency of stem cells, pathways in cancer, cell adhesion molecules, transcriptional misregulation in cancer, hippo signalling pathway, ECM‐receptor interaction, proteoglycans in cancer, TGF‐β signalling pathway, choline metabolism in cancer, PI3K‐Akt signalling pathway and P53 signalling pathway. On the contrary, the enriched pathways by cluster 1 and 3 did not present any close connections to the tumour formation (Figure 6E–G). According to the results of *K*‐means clustering algorithm (AOM/DSS vs. DSS), we speculated that these enriched GO and pathways in the cluster 2 were representative and included 11 circRNAs including mmu_circ_000622, mmu_circ_000635, mmu_circ_0001010, mmu_circ_0001073, mmu_circ_0001074, mmu_circ_0001075, mmu_circ_0001076, mmu_circ_0001077, mmu_circ_0001133, mmu_circ_0001527 and mmu_circ_0001845.

### 3.6 Mmu_circ_0001845 promoted the CAC transformation by activating the Wnt signalling pathway

**FIGURE 7:**
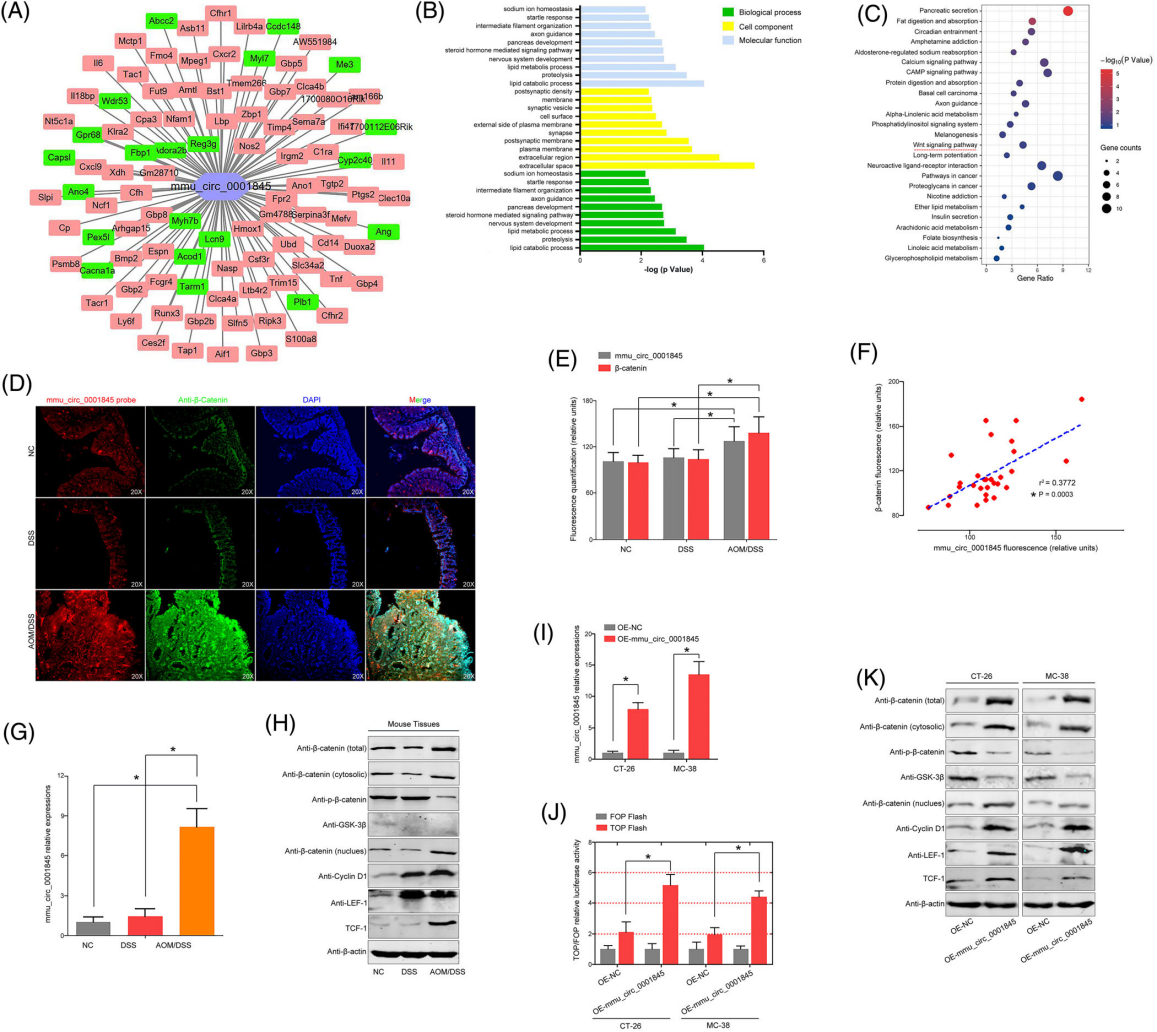
Mmu_circ_0001845 promoted the colitis‐associated cancer (CAC) transformation by activating the Wnt signalling pathway. (A) The co‐expressed genes of mmu_circ_0001845 were calculated. (B and C) The GO and KEGG analyses were done according to the mmu_circ_0001845 co‐expressed genes. (D–E) The co‐expression of mmu_circ_0001845 and β‐catenin in mice colon was determined by immunofluorescence, and the fluorescence intensity was compared. (F) The fluorescence intensity of mmu_circ_0001109 in mice was positively associated with β‐catenin. (G and H) Mmu_circ_0001845 or the Wnt‐related protein expressions in each compared group were detected by the PCR or western bolt analysis. (I–K) Mmu_circ_0001845 over‐expression vector was constructed and verified by the PCR analysis. Changes of the Wnt signalling pathway and Wnt‐related proteins were determined by the TOP/FOP flash and western blot analysis (*p < .05)

To gain insight into mechanisms of colitis‐to‐carcinoma transition during the mouse CAC, we next focused on these 11 circRNAs as calculated in cluster 2. The co‐expressed genes for each circRNA were calculated, but only mmu_circ_0001845 was proved to positively correlate with the Wnt signalling pathway through the co‐expression analysis and GO/KEGG enrichment (Figure 7A–C), while the other 10 mmu_circRNAs were not associated with the cancer‐related pathways (Figure S5A‐J). Then the co‐locations between mmu_circ_0001845 and β‐catenin were observed in all three comparisons by the FISH assays. Comparing the fluorescence intensity, we found that mmu_circ_0001845 and β‐catenin were significantly up‐regulated in the mouse colons of AOM/DSS group, compared to the NC and DSS groups (Figure 7D,E). Meanwhile, the mmu_circ_0001845 expressions in mice tissues positively associated with the protein expressions of β‐catenin (Figure 7F). Similarly, we also performed the PCR and western blot analyses to detect the expressions of these two molecules in mice tissues, and the data was consistent with our observations of FISH assays (Figure 7G,H). To reveal the potential regulatory relationships between mmu_circ_0001845 and the Wnt signalling pathway, we up‐regulated the mmu_circ_0001845 expressions in murine colon cancer cells (Figure 7I). The subsequent TOP/FOP flash and western blot analysis all strongly implied that mmu_circ_0001845 could effectively activate the Wnt signalling pathway in vitro (Figure 7J–K). These data elucidated that mmu_circ_0001845 could promote the inflammation‐to‐carcinoma transformation of CAC in mice by activating the Wnt signalling pathway.

### 3.7 Clinical significance of human RTEL1‐derived circRNAs

**FIGURE 8:**
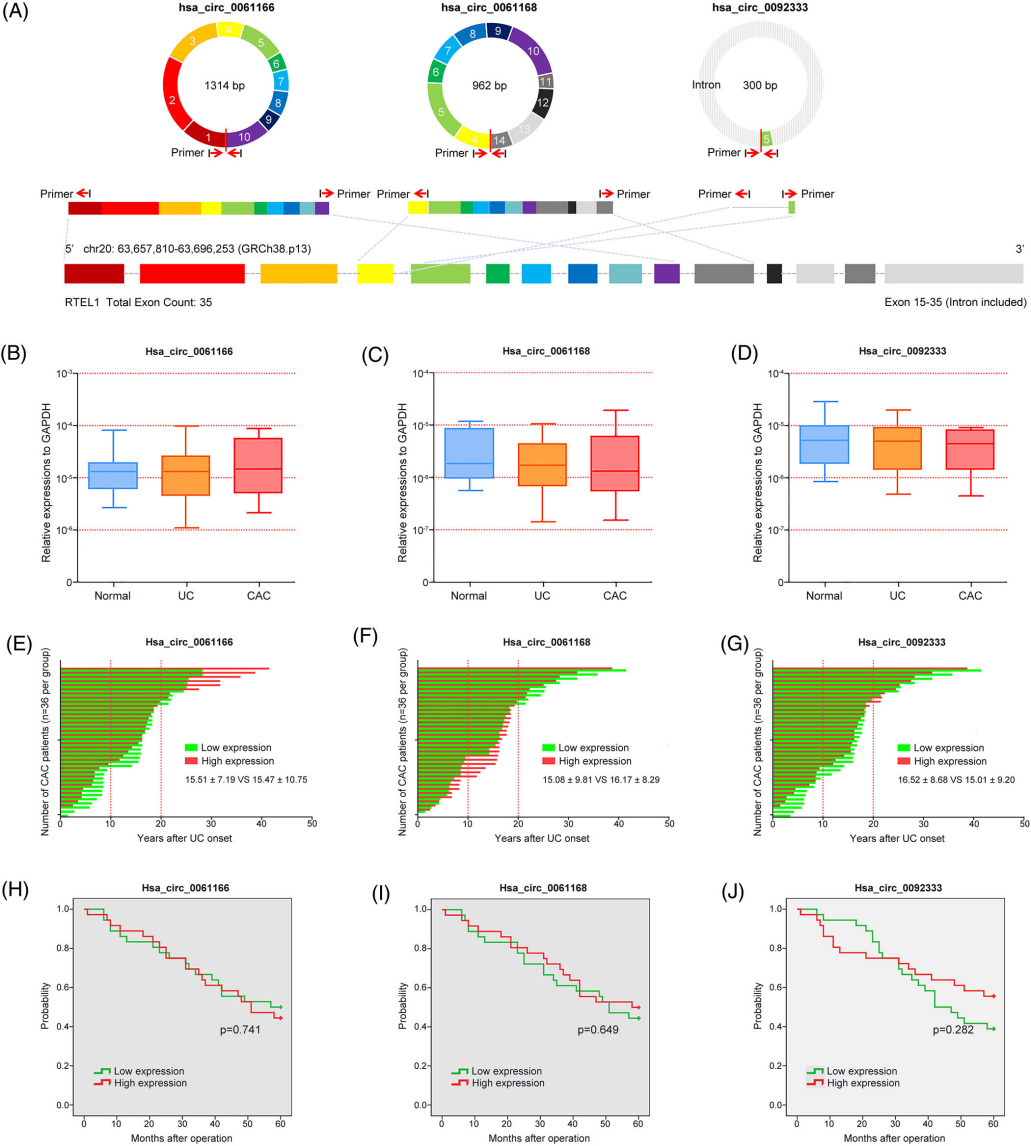
Clinical significance of human RTEL1‐derived circRNAs in ulcerative colitis (UC) and colitis‐associated cancer (CAC) patients. (A) The head‐to‐tail spicing structures of human RTEL1‐derived circRNAs were described. (B–D) The expressions of 3 RTEL1‐derived circRNAs in healthy controls, UC and CAC patients were determined by PCR analysis. (E–G) The bar plots showed the duration between the ulcerative colitis onset and time of surgery in CAC patients according to the circRNA levels. (H–J) Survival curves using the Kaplan–Meier method compared the 5‐year overall survival (OS) rates (*p < .05)

To screen the potential and novel biomarkers of inflammation or colitis‐to‐carcinoma transition in humans, we identified three homeotic circRNAs of mmu_circ_0001109 (hsa_circ_0061166, hsa_circ_0061168 and hsa_circ_0092333) from the same host gene RTEL1. These circRNAs consisted of the head‐to‐tail spicing structures from different exons and introns of human RTEL1 transcript (Figure 8A). Then a total of 86 healthy controls, 56 UC patients and 72 CAC patients were enrolled in this study. We attempted to measure the levels of these three RTEL1‐derived circRNAs in human tissues with specific divergent primers in PCR analysis, but we were unable to obtain any expressing changes in all three comparisons (Figure 8B–D). Among these 72 CAC patients, who received the colon resections, the bar plots showed the duration between the ulcerative colitis onset and time of surgery. These human RTEL1‐derived circRNAs did not influence the time of colitis‐to‐carcinoma transformation (Figure 8E–G). Besides, these 72 CAC patients entered a follow‐up of 5 years after surgery, and the data showed that up‐regulations of these RTEL1‐derived circRNAs did not lead to a poorer OS rate (Figure 8H–J). The cross‐species genomic sequence comparisons between mice and humans were performed, and the results were also very disappointing, in which the human RTEL1‐derived circRNAs were completely distinguished from mmu_circ_0001109 with only 42.68%, 56.69% or 50.96% of sequence similarities (Table S5).

### 3.8 Tumour promoting role of human PRKAR2A‐derived circRNAs

**FIGURE 9:**
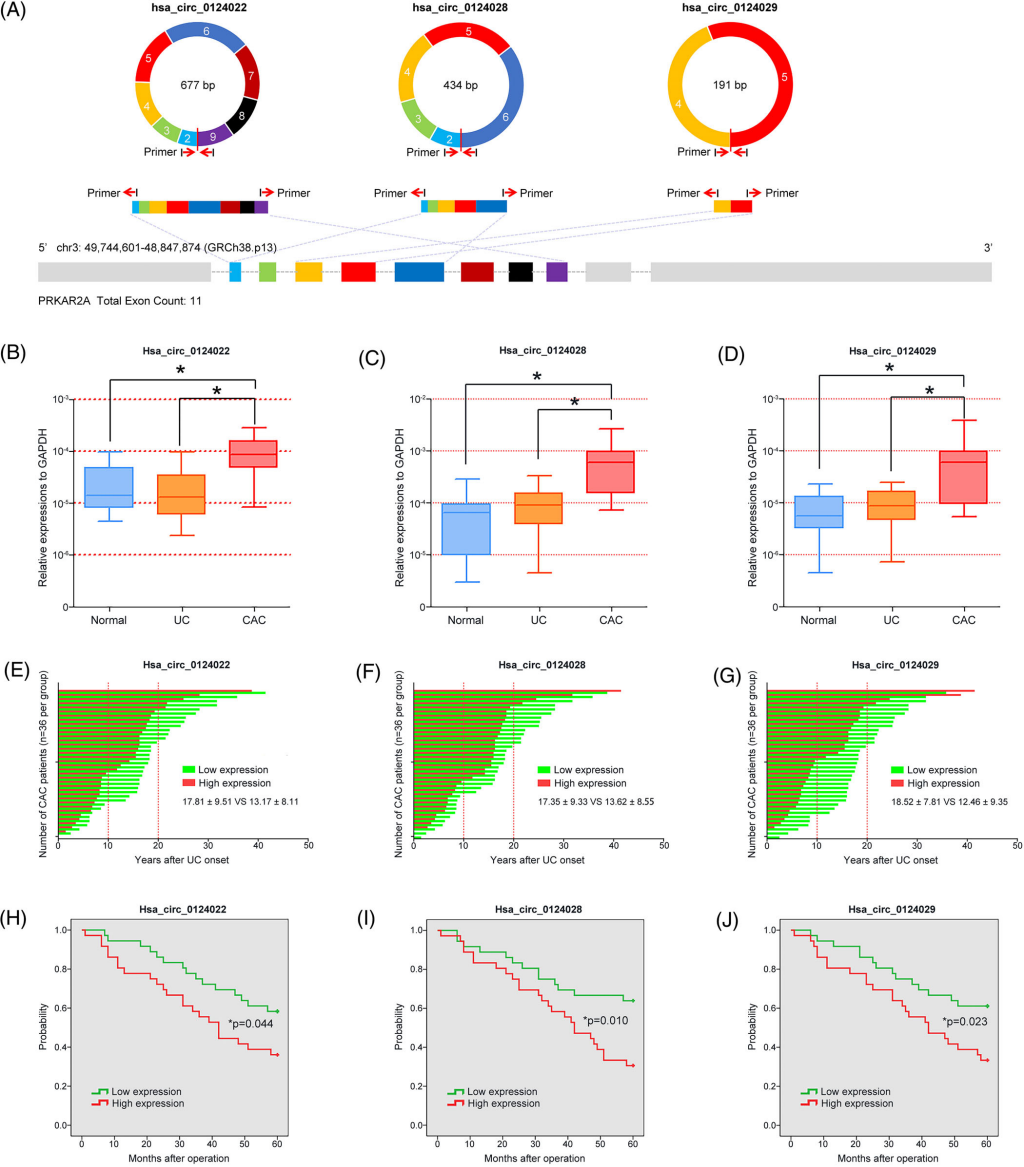
Clinical significance of human PRKAR2A‐derived circRNAs in ulcerative colitis (UC) and colitis‐associated cancer (CAC) patients. (A) The head‐to‐tail spicing structures of human PRKAR2A‐derived circRNAs were described. (B–D) The expressions of three PRKAR2A‐derived circRNAs in healthy controls, UC and CAC patients were determined by PCR analysis. (E–G) The bar plots showed the duration between the ulcerative colitis onset and time of surgery in CAC patients according to the circRNA levels. (H–J) Survival curves using the Kaplan‐Meier method compared the 5‐year overall survival (OS) rates (*p < .05)

Similarly, we also tried to search the homeotic circRNAs of mmu_circ_0001845 in humans, and a total of 21 human PRKAR2A‐derived circRNAs were identified through the RNA blasting. Among them, the RNA sequences of hsa_circ_0124022, hsa_circ_0124028 and hsa_circ_0124029 were 91.01%, 91.01% and 91.10% identical to that of mmu_circ_0001845, respectively (Table S6). Analyzing the structures of these three circRNAs, all of them derived from their host gene PRKAR2A, and also consisted of the head‐to‐tail spicing structures from exon 2–9, exon 2–6 and exon 3–4 of PRKAR2A transcript, respectively (Figure 9A). Based on the similarities of RNA sequence to mmu_circ_0001845, we speculated that these three human‐derived circRNAs were representative and might played a similar role of mmu_circ_0001845 as described in mice. Then we determined their expressions in human tissue samples by PCR analysis, and found that the levels of hsa_circ_0124022, hsa_circ_0124028 and hsa_circ_0124029 in CAC patients were significantly higher than those in UC patients or healthy controls (Figure 9B–D). More interestingly, according to the history, CAC patients with high expressions of hsa_circ_0124022, hsa_circ_0124028 or hsa_circ_0124029 all had a relative shorter duration from the UC onset to carcinoma, compared to patients with low circRNA levels (Figure 9E–G). According to the circRNA expressions in CAC patients, subgroups were divided, and the relationships between circRNAs and clinical characteristics of 72 CAC patients were analyzed. Our data showed that these human PRKAR2A‐derived circRNAs were associated with the age at surgery (hsa_circ_0124028, *p* = 0.004), disease duration (hsa_circ_0124022, *p* = 0.003; hsa_circ_0124028, *p* = 0.000; hsa_circ_0124029, *p* = 0.002) and TNM stage (hsa_circ_0124028, *p* = 0.002; hsa_circ_0124029, *p* = 0.042) (Table S7). Besides, CAC patients with high expressions of hsa_circ_0124022, hsa_circ_0124028 or hsa_circ_0124029 had an adverse clinical outcome, in comparison to the patients of low circRNA levels (Figure 9H–J). These findings strongly indicated that human PRKAR2A‐derived circRNAs might shorten the colitis‐to‐carcinoma process and cause a poor prognosis of CAC patients.

To discuss the effects of PRKAR2A‐derived circRNAs on the Wnt signalling pathway, we firstly detected the circRNA expressions in different CRC cells (Figure S6A–C), and HCT116 and KM12 cells were chosen for further experiments. Then the transfection efficiency was assessed by the PCR analysis (Figure S6D–E). Similar to the activated role of mmu_circ_0001845 in murine colon cancer cells, the results of FOP/FOP flash also confirmed that human PRKAR2A‐derived circRNAs could significantly activate the Wnt signalling in CRC cells (Figure S6F–G).

## 4 DISCUSSION

AOM, which has been proved to cause O6‐methylguanine formation and induce polypoid tumours in rodents, is now considered as a potential inducer of CRC.^28^ Repeated administrations of DSS have been now used to induce chronic colitis. When DSS is given after AOM administration, CRC carcinogenesis will be highly promoted.^29^, ^30^, ^31^ In this study, we established the mouse colitis and CAC models, and the histological examinations revealed that oral DSS administration for 7 days could induce goblet cell and crypt loss, and severe inflammatory infiltrate in C57BL/6 mice, while AOM/DSS treatment led to submucosal carcinomas with chronic inflammations. To investigate the disease‐related processes, the RNA‐seq was employed to screen the differentially expressed molecules (circRNAs and mRNAs) at the colitis and CAC stages. A total of 17 circRNAs and 679 mRNAs were involved in the mice colitis, while 116 circRNAs and 1960 mRNAs closely correlated with the inflammation‐to‐CAC transition.

Then the GO/KEGG analyses from differentially expressed mRNAs were calculated, and the results demonstrated that a cascade of pro‐inflammatory cytokines and mediators, were main regulators of the adaptive and innate immune responses. These molecules modulated crucial biological cellular functions that might trigger some downstream signalling pathways, such as the TNF signalling pathway, Chemokine signalling pathway, Toll‐like receptor signalling pathway, Jak‐STAT signalling pathway and NF‐kappa B signalling pathway. Among these signalling pathways that are involved in colonic inflammation, the Jak/STAT3 and NF‐κB signalling pathway are central. For example, the Jak/STAT signalling was found to control some key events such as over‐secretion of cytokines, cell proliferation and differentiation, and apoptosis in IBD progression.^32^, ^33^ The inhibition of JAKs and subsequent STATs by tofacitinib represented a new therapeutic strategy in IBD, as clinical trials had led to the approval of tofacitinib for UC and hinted at a possible efficacy for CD.^34^ Recently, activation of NF‐κB had been shown to play a significant role in intestinal inflammation, and the inducers of NF‐κB included IL‐1, TNF‐α, lipopolysaccharide, oxidative stress and viral products.^35^ Presently, interfering the NF‐κB pathway has been proved to be a main treatment of the UC disease. In 2020, in accordance with traditional Chinese medicinal functions, Lu et al. concluded that the characteristics of Chinese medicines targeting NF‐κB pathway might account for a primary and essential status in the amelioration of UC.^36^ A study from Greten et al. had also revealed that the intestinal inflammation induced by DSS and CAC could be attenuated by the inactivation of IKKβ (a protein kinase which could activate the NF‐κB signals) in mice.^37^ In 2012, Tang et al. dissected the changes of potential signalling pathways during the process from colitis to CAC, and found that the Wnt/β‐catenin signalling was hyperactive, which could bridge the gap between intestinal inflammation and carcinoma.^38^ In 2013, Barrett et al. reported that the Wnt signalling, which was essential to the IBD‐related intestinal stem cell programs or injury recovery processes, could also be activated in the inflammation‐to‐carcinoma transition.^39^ Consistent with the results of Li's report,^40^ our KEGG enrichment from the differentially expressed mRNAs also implied that the Wnt/β‐catenin signalling pathway participated in the development and progression of CAC. These findings might suggest that inflammation somehow caused somatic alterations of the genome and inflammatory‐related signals, and some of these alterations combined with the activated Wnt signalling pathway subsequently drove the CAC development. However, the identification and roles of circRNAs during mice colitis and colitis‐to‐carcinoma transition have still not been elucidated.

Fortunately, the *K*‐means clustering algorithm, which could effectively package circRNAs and their corresponding DEGs into clusters, provided us with a novel insight. Using this method, we divided the differentially expressed circRNAs and genes into different clusters, and each cluster was conducted with the GO and KEGG analysis, respectively. Taking the comparison between the NC and DSS groups for example, the GO and KEGG analyses from the cluster 2 of mmu_circ_0001109 and 469 mRNAs enriched most of the inflammation‐related biological process and signalling pathways, such as Chemokine signalling pathway, Cytokine‐to‐cytokine receptor interaction, TNF signalling pathway, Jak‐STAT signalling pathway, IBD, Toll‐like receptor signalling pathway and NF‐kappa B signalling pathway. Thus, we speculated that mmu_circ_0001109 might be a central mediator of mice colitis. We also constructed the co‐expression network of mmu_circ_0001109, and these GO and KEGG analyses re‐confirmed the pro‐inflammatory effects of mmu_circ_0001109. The subsequent experiments in vivo and in vitro including the co‐focal examinations, PCR and western blot analyses, all supported our hypothesis that mmu_circ_0001109 up‐regulation could significantly activate the Jak/STAT and NF‐kappa B signalling pathways in mice. The status of high mmu_circ_0001109 expression and activated Jak/STAT and NF‐kappa B signalling pathways were prolonged during the whole process, from colitis to the final carcinoma formation. Meanwhile, a similar *K*‐means clustering algorithm was also performed between the DSS and AOM/DSS groups. Among 116 differentially expressed circRNAs, 11 ones were identified to potentially associate with the Wnt signalling pathway. We also constructed the co‐expression networks for each circRNAs, and revealed that only mmu_circ_0001845 might be closely associated with the colitis‐to‐carcinoma transition via the Wnt signalling pathway. Fortunately, the results in vivo including the co‐focal examinations observation, PCR and western blot analyses also confirmed our predictions from the bioinformatics analysis. Mmu_circ_0001845 was significantly up‐regulated in AOM/DSS‐induced CAC tissues, and positively associated with the expressions of β‐catenin. Besides, over‐expression of mmu_circ_0001845 in murine colon cancer cells could also effectively activate the Wnt signalling pathway in vitro. Thus, we speculated that circRNAs might play different roles in CAC formation, in which mmu_circ_0001109 could induce the persistent inflammation, whereas mmu_circ_0001845 could cause the colitis‐to‐carcinoma transition through the Wnt signalling pathway.

Since the positive selections of circRNAs in colitis and CAC, we wondered if the homeotic circRNAs of mmu_circ_0001109 or mmu_circ_0001845 also played a same role during the IBD or CAC process in humans. But there is no existing tool for us to visualize and explore the homeotic circRNAs across different species and tissues.^41^ Emerging evidence shows that most of the intronic miRNAs are processed by the microprocessor, which is similar to the manners from the pre‐mRNA molecules to mRNAs.^42^, ^43^, ^44^ CircRNAs, which are considered as an abundant and diverse class of non‐canonical and hyper‐stable RNAs, may arise from an alternative splicing (AS) form of back‐splicing. Splicing can be considered as a major event in the processing of non‐coding RNAs from the host transcript. CircRNAs are also proved to generate from the pre‐mRNAs through a back‐splicing mechanism.^45^, ^46^ These phenomena might give us a clue to search the potential homeotic circular RNAs across different species. RTEL1 is a component of DNA repair and telomere maintenance machineries. Recent studies revealed that RTEL1 might act as a cancer susceptibility gene and implicated in a number of telomere dysfunction syndromes.^47^, ^48^ However, no reports implied the direct or indirect associations of RTEL1 with sporadic colorectal cancer or CAC. In this study, we attempted to measure the levels of these three RTEL1‐derived circRNAs in a total of 86 healthy controls, 56 UC patients and 72 CAC patients, but no expressing changes of any circRNAs in all three comparisons were observed. More importantly, the data also showed that there were no positive associations of human RTEL1‐derived circRNAs with the time of colitis‐to‐carcinoma transformation and the 5‐year OS of CAC patients. Then the cross‐species genomic sequence comparisons between mice and humans were performed, but the results were so disappointing that the human RTEL1‐derived circRNAs were completely distinguished from mmu_circ_0001109 with only 42.68% (hsa_circ_0061166), 56.69% (hsa_circ_0061168) or 50.96% (hsa_circ_0092333) of sequence similarities. The low percentage of matched sequence might partly explained why human RTEL1‐derived circRNAs did not play any promoting role in the UC and CAC development.

PRKAR2A is a ligand of Prokineticin1, which can transmit multiple intracellular signals to induce some functional changes.^49^ Through immunohistochemical staining, PRK2R2A expression was observed mainly on the cell membrane of the primary lesion in 147 of (45.3%) 324 human CRC cases. Patients of over‐expressed PRKAR2A led to a relatively lower 5‐year survival rate and higher recurrence.^50^ In this study, similar blasting analyses were also performed between 21 human PRKAR2A‐derived circRNAs and mmu_circ_0001845, and the matched percentages of has_circ_0124022, has_circ_0124028 and has_circ_0124029 were consistently more than 90%. We speculated that these three circRNAs might be the key molecules during the CAC transformation. Interestingly, the clinical data showed that these 3 PRKAR2A‐derived circRNAs were all significantly up‐regulated in CAC tissues, compared to those tissues of UC and normal colon mucosa tissues. Meanwhile, CAC patients with high hsa_circ_0124022, hsa_circ_0124028 or hsa_circ_0124029 expressions had a relatively shorter duration from the UC onset to carcinoma, compared to patients of low circRNA expressions, which also led to an unsatisfactory clinical outcome. Though the tumour‐promoting roles and mechanisms had not been fully elucidated, our findings strongly revealed that human PRKAR2A‐derived circRNAs might shorten the colitis‐to‐carcinoma process and cause a poor prognosis of CAC patients.

To our knowledge, this is the first study to evaluate the potential role of circRNAs in the established mouse colitis and CAC models. We screened the differentially expressed circRNAs and mRNAs at the different states of CAC, and the bioinformatics analyses including the GO/KEGG enrichment, *K‐*means clustering algorithm and co‐expression networks, all implied that mmu_circ_00001109 and mmu_circ_0001845 might play a promoting role during the inflammation or colitis‐to‐carcinoma transition. The results in vivo and in vitro also confirmed that mmu_circ_00001109 could significantly accelerate the colitis through activating the Jak/STAT and NF‐kappa B signalling pathways, while mmu_circ_0001845 closely associated with the Wnt signalling pathway. By RNA blasting between mice and humans, the RTEL1‐ and PRKAR2A‐derived circRNAs were identified, and the data showed that RTEL1‐derived circRNAs had no clinical significance in human colitis and CAC. However, three PRKAR2A‐derived circRNAs, which had high RNA similarities of mmu_circ_0001845, were remarkably up‐regulated in CAC tissues and promoted the colitis to CAC transition. Our results suggested that these human PRKAR2A‐derived circRNAs could be served as novel candidates for distinguishing CAC patients and predicted the prognosis of CAC. Additionally, targeting these specific human circRNAs might also offer promising therapeutic strategies for CAC.

## CONFLICT OF INTEREST

The authors declare that they have no conflict of interest.

## Supporting information

Figures S1–S6

Tables s1‐s7
