# Supplementary material for: PRKAR2A‐derived circular RNAs promote the malignant transformation of colitis and distinguish patients with colitis‐associated colorectal cancer
Source: Clin Transl Med. 2022 Feb 20;12(2):e683. doi: 10.1002/ctm2.683 (PMC8858608; doi:10.1002/ctm2.683)
Supplement: Supplementary file 1 — Figures S1–S6 [file CTM2-12-e683-s001.docx]

# **PRKAR2A-derived circular RNAs** promote **the malignant transformation of colitis and distinguish patients with [colitis-associated colorectal](https://www.geenmedical.com/article?id=33461100&type=true)cancer**

Daiwei Wan^1, *^, Sentai Wang^1, *^, Zhihua Xu^1, *^, Xinquan Zan^1^, Fei Liu^2^,

Ye Han^1^, Min Jiang^3, #^, Airong Wu^2, #^, Qiaoming Zhi^1, #^

**Supplementary Figures**

Supplementary Fig. 1

**
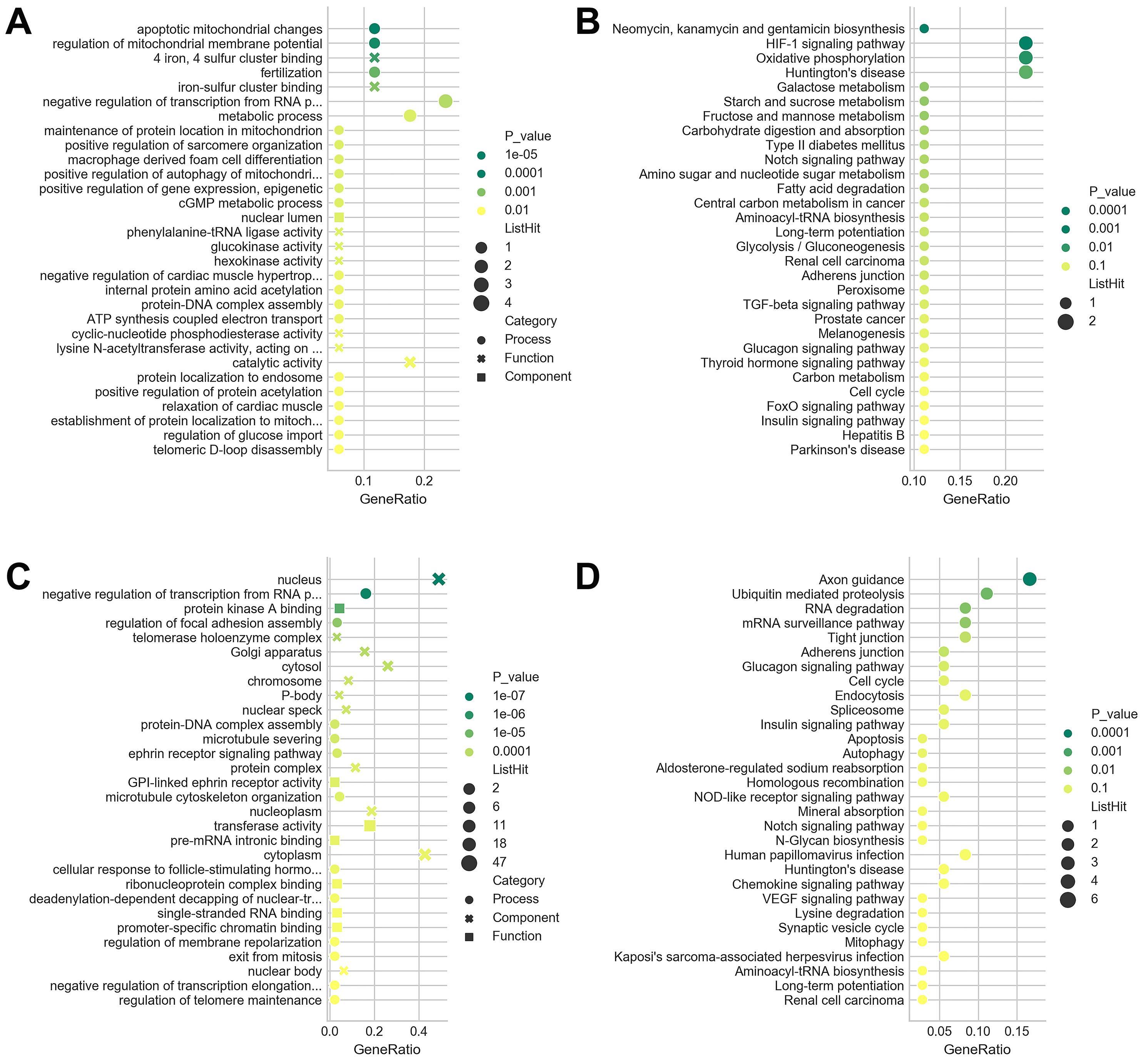
**

**Supplementary Fig. 1** The GO and KEGG analysis according to the parental genes of circRNAs (DSS vs. NC or AOM/DSS vs. DSS) predicted the most enriched terms and signaling pathways. (A-B) The GO analysis was calculated, and the top 10 biological processes, cell components and molecular functions were presented. (C-D) The KEGG analysis described the most enriched signaling pathways.

Supplementary Fig. 2


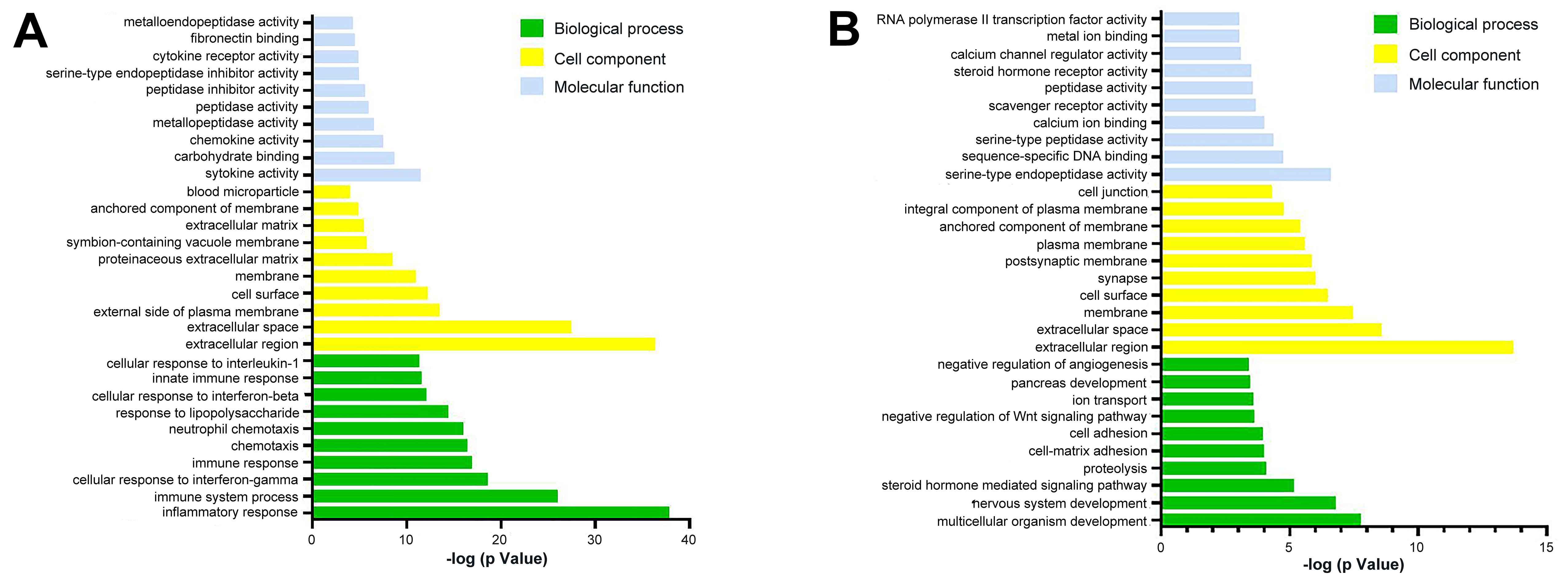


**Supplementary Fig. 2** (A) The GO analysis from differentially expressed mRNAs (DSS vs. NC) was calculated, and the top 10 biological processes, cell components and molecular functions were presented. (B) The GO analysis from differentially expressed mRNAs (AOM/DSS vs. DSS) also presented the top 10 biological processes, cell components and molecular functions.

Supplementary Fig. 3

**
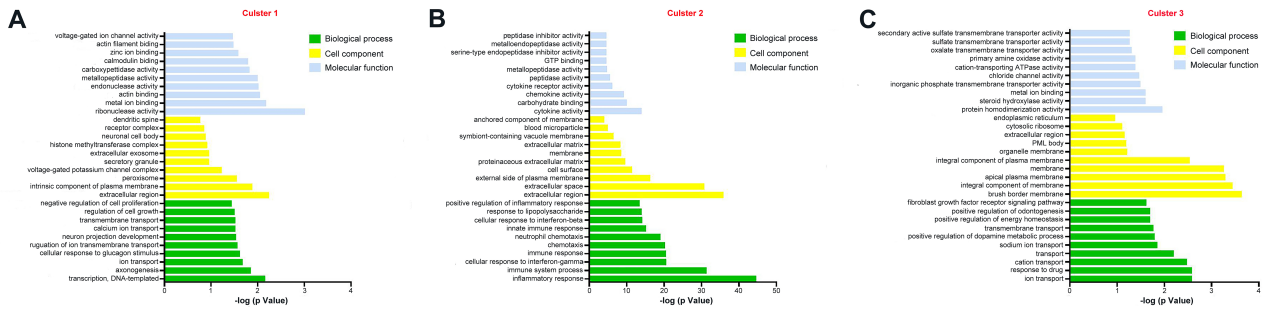
**

**Supplementary Fig. 3** The GO analyses from differentially expressed mRNAs (DSS vs. NC) in cluster 1-3 were calculated.

Supplementary Fig. 4

**
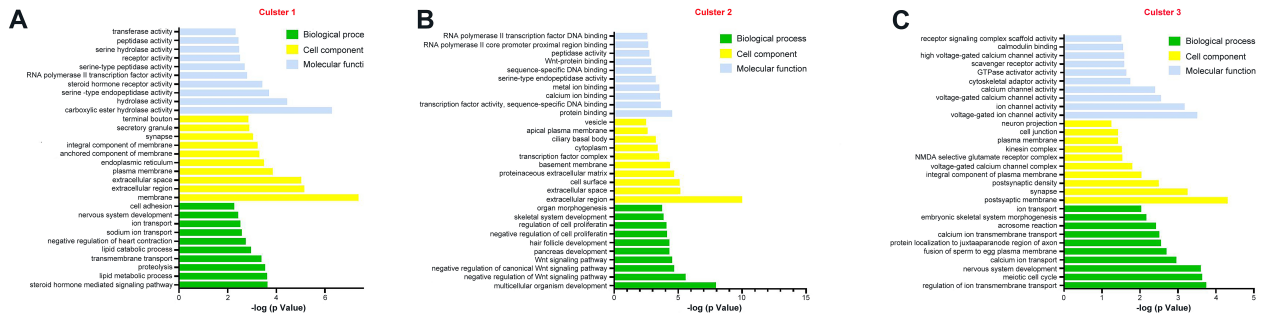
**

**Supplementary Fig. 4** The GO analyses from differentially expressed mRNAs (AOM/DSS vs. DSS) in cluster 1-3 were calculated.

Supplementary Fig. 5

**
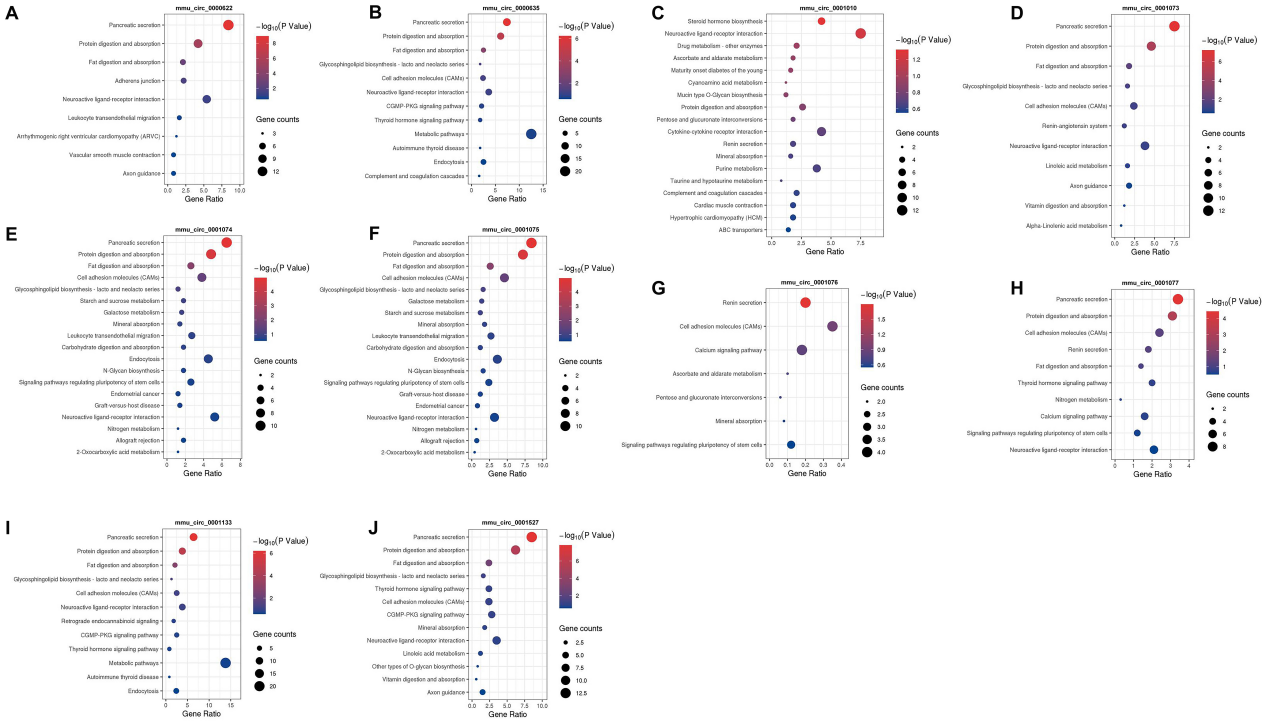
**

**Supplementary Fig. 5** The KEGG analyses from other 10 circRNAs in cluster 2 (AOM/DSS vs. DSS) were calculated according to their corresponding co-expressed genes.

Supplementary Fig. 6


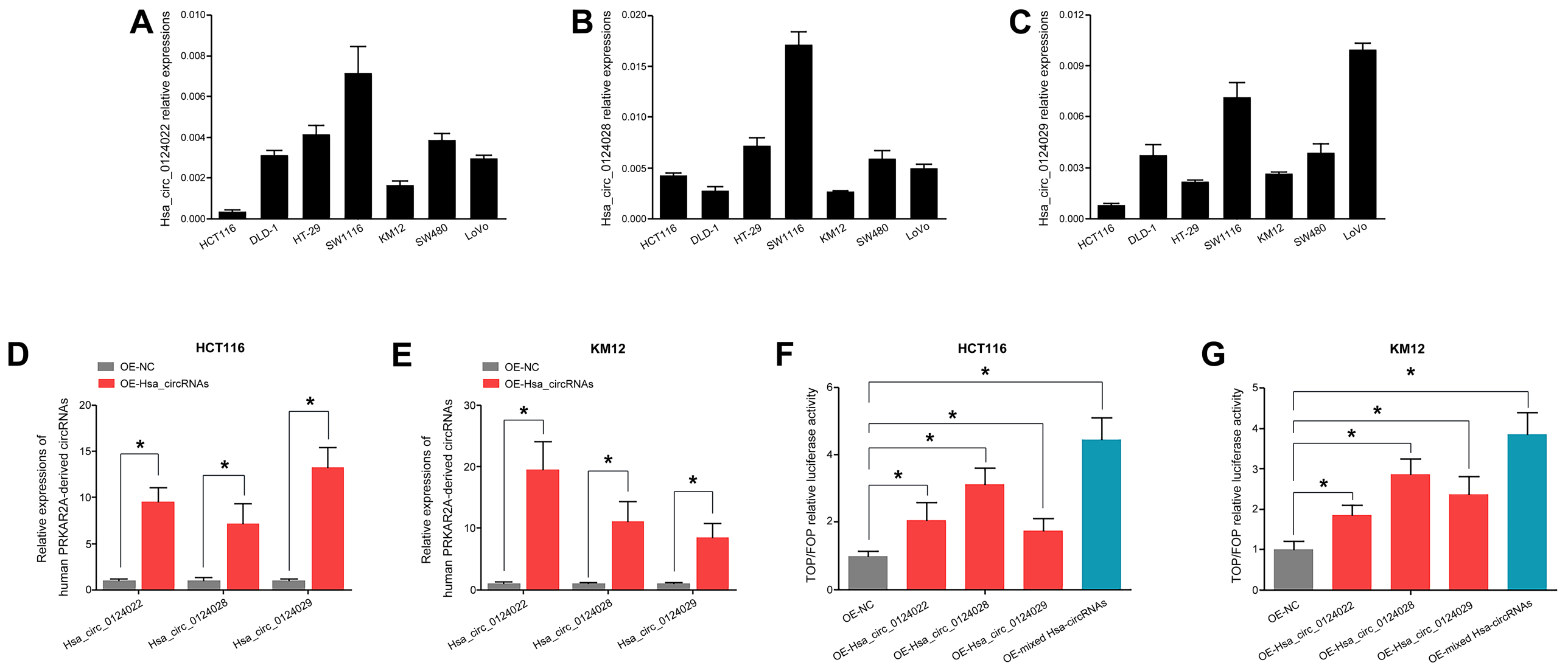


**Supplementary Fig. 6** Human PRKAR2A-derived circRNAs could activate the Wnt signaling pathway in CRC cells. (A-C) Hsa_circ_0124022, hsa_circ_0124028 and hsa_circ_0124029 expressions were determined in different CRC cells by the PCR analysis. (D-E) The over-expressing transfection efficiency was verified by the PCR analysis. (F-G) The results of FOP/FOP flash confirmed that over-expressing hsa_circ_0124022, hsa_circ_0124028, hsa_circ_0124029 or mixed circRNAs could significantly activate the Wnt signaling pathway in CRC cells. (*P<0.05)
