# Supplementary material for: PRKAR2A‐derived circular RNAs promote the malignant transformation of colitis and distinguish patients with colitis‐associated colorectal cancer
Source: Clin Transl Med. 2022 Feb 20;12(2):e683. doi: 10.1002/ctm2.683 (PMC8858608; doi:10.1002/ctm2.683)
Supplement: Supplementary file 2 — Tables s1‐s7 [file CTM2-12-e683-s002.doc]

**Supplementary Table 1 Demographic and clinical characteristics of UC and CAC patients**

| **Variable** | **UC (n=56)** | **UC with CAC (n=72)** |
| --- | --- | --- |
| Gender, male | 32 | 47 |
| Age at UC onset (years) | 36.85±15.64 | 32.74±13.12 |
| Age at CAC detection (years) | - | 48.23±15.87 |
| Smoking habit | 33 | 28 |
| History of appendectomy | 5 | 7 |
| UC extent |  |  |
| Pancolitis (E3) | 31 | 58 |
| Left side colitis (E2) | 19 | 13 |
| Proctitis (E1) | 6 | 1 |
| Preoperative therapy (aminosalicylic acid, hormones and/or immuno-suppressants) | 38 | 68 |
| Type of surgery |  |  |
| Total colectomy | 21 | 67 |
| Other colon surgery | 3 | 5 |
| Endoscopic biopsy/resection | 32 | 0 |
| Disease duration (Period between UC onset and surgery, years) | - | 15.49±9.08 |
| Cancer stage |  |  |
| I/II | - | 23 |
| III/IV | - | 49 |

**Supplementary Table 2** Primers used for circRNA expression analysis

| **CircRNA ID** | **Sense** | **Anti-sense** | **Product size** |
| --- | --- | --- | --- |
| mmu_circ_0001109 | CCACCGGAGAGCAGCTAAG | AGATACCCAGCCCCACCAG | 120 |
| mmu_circ_0001845 | CGACGGGGACAACTTTTATG | TGCTAGGAACCGGAACAATC | 199 |
| hsa_circ_0061166 | ATAGACCAGGTGCTGGAGGA | TCAGGAACTCAACCGACTCC | 195 |
| hsa_circ_0061168 | GACCAGATCATCCAGCACCT | ACCTGTGTGAGTTGCGAGTG | 148 |
| hsa_circ_0092333 | GCGTGTCTCCATACAGCTCA | ACCCGACAGAACACCAAGAC | 156 |
| hsa_circ_0124022 | ACTCAGGGTGAAAAGGCTGA | GGATGAATCACCCTTGGATCT | 172 |
| hsa_circ_0124028 | ATGTACAACACCCCGAGAGC | GGATGAATCACCCTTGGATCT | 200 |
| hsa_circ_0124029 | TTCTCGATGCCATGTTTGAA | CAAGCTTCCTGAAGTCTGCAT | 141 |
| GAPDH | TGTTGCCATCAATGACCCCTT | CTCCACGACGTACTCAGCG | 202 |

Supplementary Table 3 The information of differential expressed circRNAs (DSS vs. NC)

| **Target ID** | **Fold Change** | **Regulation** | **Chromosome** | **Start** | **End** | **Length** | **Gene Symbol** |
| --- | --- | --- | --- | --- | --- | --- | --- |
| mmu_circ_0000044 | -2.036260681 | down | chr1 | 63194969 | 63195145 | 176 | Ndufs1 |
| mmu_circ_0000382 | -2.207581144 | down | chr12 | 56760034 | 56778252 | 1214 | Ralgapa1 |
| mmu_circ_0000450 | -2.021421013 | down | chr13 | 35044849 | 35072220 | 17177 | Eci3 |
| mmu_circ_0000453 | -2.074052238 | down | chr13 | 36323990 | 36338363 | 292 | Fars2 |
| mmu_circ_0000553 | -2.048745194 | down | chr14 | 72989560 | 72998949 | 315 | Fndc3a |
| mmu_circ_0000620 | -2.551766184 | down | chr15 | 78945084 | 78945601 | 191 | Micall1 |
| mmu_circ_0000622 | -2.213081205 | down | chr15 | 79658068 | 79658179 | 111 | Cbx6 |
| mmu_circ_0000626 | -2.355928515 | down | chr15 | 81462043 | 81462942 | 329 | Ep300 |
| mmu_circ_0000821 | -2.299762783 | down | chr17 | 81053201 | 81055261 | 169 | Map4k3 |
| mmu_circ_0001109 | 2.232988304 | up | chr2 | 181081536 | 181081799 | 157 | Rtel1 |
| mmu_circ_0001165 | -2.677971863 | down | chr3 | 122450806 | 122451395 | 589 | Pde5a |
| mmu_circ_0001171 | -2.112412153 | down | chr3 | 132983899 | 132993476 | 284 | Ppa2 |
| mmu_circ_0001286 | -2.385398226 | down | chr4 | 137790157 | 137793222 | 251 | Hp1bp3 |
| mmu_circ_0001485 | -2.145773402 | down | chr6 | 82680761 | 82689668 | 1344 | Hk2 |
| mmu_circ_0001527 | -3.609985945 | down | chr6 | 131318489 | 131318573 | 84 | Ybx3 |
| mmu_circ_0001764 | -2.025099891 | down | chr9 | 31989426 | 31997646 | 432 | Arhgap32 |
| mmu_circ_0001813 | -2.132942989 | down | chr9 | 77064783 | 77078836 | 14053 | Mlip |

Supplementary Table 4 The information of differential expressed circRNAs (AOM/DSS vs. DSS)

| **Target ID** | **Fold Change** | **Regulation** | **Chromosome** | **Start** | **End** | **Length** | **Gene Symbol** |
| --- | --- | --- | --- | --- | --- | --- | --- |
| mmu_circ_0000016 | -3.428500631 | down | chr1 | 22336373 | 22369621 | 516 | Rims1 |
| mmu_circ_0000018 | -3.4607911 | down | chr1 | 22336373 | 22387644 | 629 | Rims1 |
| mmu_circ_0000025 | -2.760532032 | down | chr1 | 36200363 | 36200445 | 82 | Uggt1 |
| mmu_circ_0000026 | -2.54630043 | down | chr1 | 36911688 | 36946170 | 296 | Tmem131 |
| mmu_circ_0000066 | -2.027908424 | down | chr1 | 120316016 | 120316495 | 479 | Clasp1 |
| mmu_circ_0000118 | -2.440241491 | down | chr1 | 180260045 | 180260360 | 90 | Hnrnpu |
| mmu_circ_0000137 | -2.40954408 | down | chr1 | 196844794 | 196850600 | 904 | A330023F24Rik |
| mmu_circ_0000159 | -2.533659413 | down | chr10 | 39974069 | 39974270 | 109 | Gtf3c6 |
| mmu_circ_0000174 | -2.570806868 | down | chr10 | 68211123 | 68214076 | 335 | Tmem26 |
| mmu_circ_0000181 | -2.024929499 | down | chr10 | 75147036 | 75162840 | 629 | Cabin1 |
| mmu_circ_0000207 | -3.597528165 | down | chr10 | 92684331 | 92701788 | 991 | Cdk17 |
| mmu_circ_0000212 | -2.6821905 | down | chr10 | 115954372 | 115986231 | 333 | Cnot2 |
| mmu_circ_0000217 | -2.246905337 | down | chr10 | 119513488 | 119513597 | 109 |  |
| mmu_circ_0000240 | -3.156757592 | down | chr11 | 20603713 | 20609749 | 307 | Aftph |
| mmu_circ_0000242 | -3.268949911 | down | chr11 | 20625687 | 20627642 | 1955 | Aftph |
| mmu_circ_0000261 | -2.340105082 | down | chr11 | 53182161 | 53189135 | 952 | Aff4 |
| mmu_circ_0000271 | -3.551989479 | down | chr11 | 61959807 | 61975412 | 586 | Specc1 |
| mmu_circ_0000282 | -2.68997148 | down | chr11 | 70688247 | 70700673 | 494 | Rabep1 |
| mmu_circ_0000288 | -3.020921334 | down | chr11 | 74742494 | 74803717 | 2778 | Smg6 |
| mmu_circ_0000291 | -3.026980273 | down | chr11 | 75305463 | 75305539 | 76 | Prpf8 |
| mmu_circ_0000307 | -2.05866795 | down | chr11 | 86960435 | 86962865 | 192 | Trim37 |
| mmu_circ_0000317 | -2.491687003 | down | chr11 | 97091143 | 97099572 | 505 | Npepps |
| mmu_circ_0000323 | -2.484575574 | down | chr11 | 101156283 | 101160390 | 550 | Becn1 |
| mmu_circ_0000371 | -2.15146977 | down | chr12 | 52722766 | 52762700 | 1744 | Strn3 |
| mmu_circ_0000373 | -2.124822367 | down | chr12 | 52756388 | 52762700 | 260 | Strn3 |
| mmu_circ_0000379 | -2.23465837 | down | chr12 | 54240367 | 54243755 | 3388 | Akap6 |
| mmu_circ_0000385 | -2.08042168 | down | chr12 | 70421359 | 70423337 | 343 | Nemf |
| mmu_circ_0000403 | -3.964529614 | down | chr12 | 86390641 | 86391322 | 345 | Ylpm1 |
| mmu_circ_0000420 | -2.426751173 | down | chr12 | 112830787 | 112842775 | 246 | Mark3 |
| mmu_circ_0000421 | -3.130829148 | down | chr12 | 112830787 | 112856594 | 361 | Mark3 |
| mmu_circ_0000433 | -4.03957025 | down | chr13 | 17842090 | 17864791 | 1386 | Cdk13 |
| mmu_circ_0000434 | -2.262737324 | down | chr13 | 17854937 | 17864791 | 1139 | Cdk13 |
| mmu_circ_0000452 | -3.660945573 | down | chr13 | 36299495 | 36338363 | 24787 | Fars2 |
| mmu_circ_0000495 | -2.265373434 | down | chr13 | 104674222 | 104679365 | 364 | Erbin |
| mmu_circ_0000545 | -2.711061055 | down | chr14 | 58484357 | 58497296 | 252 | Zdhhc20 |
| mmu_circ_0000549 | -2.245798739 | down | chr14 | 65444437 | 65447505 | 271 | Hmbox1 |
| mmu_circ_0000558 | -2.49391105 | down | chr14 | 78911484 | 78911543 | 59 | Akap11 |
| mmu_circ_0000591 | -2.727059259 | down | chr15 | 38954758 | 38961844 | 407 | Dcaf13 |
| mmu_circ_0000611 | -2.816731541 | down | chr15 | 73122464 | 73134977 | 529 | Ptk2 |
| mmu_circ_0000622 | 3.516854712 | up | chr15 | 79658068 | 79658179 | 111 | Cbx6 |
| mmu_circ_0000626 | -3.40616216 | down | chr15 | 81462043 | 81462942 | 329 | Ep300 |
| mmu_circ_0000635 | 2.024868706 | up | chr15 | 88641726 | 88642589 | 328 | Alg12 |
| mmu_circ_0000658 | -2.468856977 | down | chr16 | 11104658 | 11128796 | 539 | Txndc11 |
| mmu_circ_0000685 | -3.100453198 | down | chr16 | 33421406 | 33468269 | 901 | Zfp148 |
| mmu_circ_0000695 | -3.794230773 | down | chr16 | 62827096 | 62827149 | 53 | Arl13b |
| mmu_circ_0000697 | -3.366204437 | down | chr16 | 63772735 | 63773396 | 661 | Epha3 |
| mmu_circ_0000700 | -2.623100279 | down | chr16 | 74352795 | 74353122 | 327 | Robo2 |
| mmu_circ_0000719 | -2.557624837 | down | chr16 | 95053905 | 95054826 | 921 |  |
| mmu_circ_0000798 | -2.001863154 | down | chr17 | 50745619 | 50750520 | 2691 | Plcl2 |
| mmu_circ_0000807 | -2.038012967 | down | chr17 | 66373415 | 66402431 | 994 | Ankrd12 |
| mmu_circ_0000814 | -2.999471186 | down | chr17 | 74751289 | 74758689 | 267 | Spast |
| mmu_circ_0000826 | -2.385855799 | down | chr17 | 84785850 | 84815727 | 29877 |  |
| mmu_circ_0000869 | -2.584457587 | down | chr18 | 43725516 | 43737163 | 903 | Jakmip2 |
| mmu_circ_0000882 | -2.912044043 | down | chr18 | 65157001 | 65239725 | 249 | Nedd4l |
| mmu_circ_0000889 | -2.081743101 | down | chr18 | 69621515 | 69625101 | 159 | Tcf4 |
| mmu_circ_0000926 | -3.088134282 | down | chr19 | 5797798 | 5797857 | 59 | Malat1 |
| mmu_circ_0000929 | -2.153248205 | down | chr19 | 5800853 | 5800910 | 57 | Malat1 |
| mmu_circ_0000946 | -2.128570601 | down | chr19 | 36936575 | 36946248 | 569 | Tnks2 |
| mmu_circ_0000954 | -2.833870432 | down | chr19 | 45012854 | 45022542 | 1217 | Slf2 |
| mmu_circ_0000956 | -3.354347985 | down | chr19 | 45710224 | 45715011 | 415 | Fbxw4 |
| mmu_circ_0000982 | -3.007437815 | down | chr2 | 18023084 | 18045439 | 363 | Mllt10 |
| mmu_circ_0001010 | 2.585964055 | up | chr2 | 35744784 | 35800479 | 1019 | Ttll11 |
| mmu_circ_0001021 | -2.155264324 | down | chr2 | 59786143 | 59800687 | 1276 | Baz2b |
| mmu_circ_0001047 | -4.228657003 | down | chr2 | 92054205 | 92070881 | 396 | Phf21a |
| mmu_circ_0001053 | -2.303411644 | down | chr2 | 104449027 | 104449343 | 198 | Cstf3 |
| mmu_circ_0001059 | -2.79966247 | down | chr2 | 118902671 | 118912386 | 374 | Knl1 |
| mmu_circ_0001065 | -2.360723571 | down | chr2 | 126649711 | 126651525 | 429 | Trpm7 |
| mmu_circ_0001073 | 2.522677201 | up | chr2 | 139803342 | 139883235 | 743 | Tasp1 |
| mmu_circ_0001074 | 2.828890728 | up | chr2 | 139810864 | 139883235 | 636 | Tasp1 |
| mmu_circ_0001075 | 2.004297491 | up | chr2 | 139821808 | 139883235 | 556 | Tasp1 |
| mmu_circ_0001076 | 2.632779156 | up | chr2 | 139834434 | 139883235 | 471 | Tasp1 |
| mmu_circ_0001077 | 2.820696531 | up | chr2 | 139867829 | 139883235 | 350 | Tasp1 |
| mmu_circ_0001127 | -2.564760022 | down | chr3 | 54083784 | 54106216 | 845 | Trpc4 |
| mmu_circ_0001133 | 2.02195364 | up | chr3 | 55889163 | 55895042 | 678 | Nbea |
| mmu_circ_0001143 | -2.251377272 | down | chr3 | 86336006 | 86346633 | 409 | Lrba |
| mmu_circ_0001222 | -3.41781883 | down | chr4 | 82481349 | 82500019 | 694 | Zdhhc21 |
| mmu_circ_0001232 | -2.149026743 | down | chr4 | 87767173 | 87831986 | 1229 |  |
| mmu_circ_0001249 | -2.978973177 | down | chr4 | 117255204 | 117265767 | 269 | Eri3 |
| mmu_circ_0001262 | -2.414883364 | down | chr4 | 127974429 | 128015941 | 41512 |  |
| mmu_circ_0001271 | -2.029628676 | down | chr4 | 130224839 | 130257025 | 443 | Pum1 |
| mmu_circ_0001314 | -2.335683879 | down | chr5 | 8399687 | 8405838 | 449 | Dbf4 |
| mmu_circ_0001346 | -5.000014258 | down | chr5 | 44159302 | 44159402 | 100 | Fbxl5 |
| mmu_circ_0001362 | -3.059865336 | down | chr5 | 84760252 | 84760916 | 664 | Epha5 |
| mmu_circ_0001369 | -2.22716321 | down | chr5 | 101234916 | 101241221 | 1103 | Abraxas1 |
| mmu_circ_0001371 | -2.085690685 | down | chr5 | 102397887 | 102410608 | 335 | Wdfy3 |
| mmu_circ_0001384 | -3.893243689 | down | chr5 | 107064628 | 107065342 | 714 | Zfp644 |
| mmu_circ_0001393 | -2.073124223 | down | chr5 | 115890493 | 115891454 | 201 | Msi1 |
| mmu_circ_0001395 | -2.289439331 | down | chr5 | 118542504 | 118545738 | 404 | Fbxw8 |
| mmu_circ_0001409 | -2.24543446 | down | chr5 | 124194963 | 124195598 | 250 | Rsrc2 |
| mmu_circ_0001423 | -2.599615798 | down | chr5 | 134967762 | 134976019 | 708 | Clip2 |
| mmu_circ_0001444 | -3.043598692 | down | chr6 | 17794908 | 17805028 | 569 | St7 |
| mmu_circ_0001470 | -2.476389666 | down | chr6 | 47490675 | 47527666 | 1843 | Ezh2 |
| mmu_circ_0001471 | -3.061160044 | down | chr6 | 47526534 | 47527666 | 253 | Ezh2 |
| mmu_circ_0001512 | -3.184462615 | down | chr6 | 119130082 | 119150969 | 366 | Dcp1b |
| mmu_circ_0001514 | -2.027995432 | down | chr6 | 119644588 | 119723919 | 1391 | Erc1 |
| mmu_circ_0001527 | 2.910847513 | up | chr6 | 131318489 | 131318573 | 84 | Ybx3 |
| mmu_circ_0001536 | -2.131242984 | down | chr6 | 147456305 | 147524102 | 779 | Ccdc91 |
| mmu_circ_0001570 | -2.2705182 | down | chr7 | 63352746 | 63353856 | 341 | Herc2 |
| mmu_circ_0001584 | -3.370939077 | down | chr7 | 82281183 | 82302335 | 922 | Sv2b |
| mmu_circ_0001589 | -2.489083891 | down | chr7 | 89038684 | 89050522 | 519 | Hdgfl3 |
| mmu_circ_0001598 | -3.542436785 | down | chr7 | 104809322 | 104813276 | 1976 | Rsf1 |
| mmu_circ_0001607 | -2.096610782 | down | chr7 | 109171825 | 109171927 | 102 | Rnf121 |
| mmu_circ_0001624 | -4.12349018 | down | chr7 | 128191351 | 128200913 | 586 | Mettl9 |
| mmu_circ_0001667 | -2.141465097 | down | chr8 | 26902628 | 26904264 | 185 | Lsm1 |
| mmu_circ_0001709 | -3.350130966 | down | chr8 | 88420328 | 88446141 | 305 | Phkb |
| mmu_circ_0001715 | -2.184603852 | down | chr8 | 93965561 | 93998351 | 368 | Fto |
| mmu_circ_0001732 | -2.333771222 | down | chr8 | 125906426 | 125907343 | 194 | Tcf25 |
| mmu_circ_0001737 | -3.864329275 | down | chr8 | 129894147 | 129913398 | 1177 | Pard3 |
| mmu_circ_0001746 | -2.214121179 | down | chr9 | 16179392 | 16182698 | 3306 | Fat3 |
| mmu_circ_0001761 | -2.478620375 | down | chr9 | 31959506 | 32015850 | 778 | Arhgap32 |
| mmu_circ_0001777 | -2.31257884 | down | chr9 | 57376762 | 57378169 | 239 | Cox5a |
| mmu_circ_0001811 | -2.946149791 | down | chr9 | 77012571 | 77078836 | 40637 | Mlip |
| mmu_circ_0001812 | -2.112501194 | down | chr9 | 77021765 | 77078836 | 40508 | Mlip |
| mmu_circ_0001826 | -2.586116848 | down | chr9 | 89495879 | 89498232 | 2353 | AF529169 |
| mmu_circ_0001845 | 3.504633667 | up | chr9 | 108616603 | 108635594 | 434 | Prkar2a |
| mmu_circ_0001903 | -3.101261362 | down | chrX | 156254508 | 156255230 | 722 |  |

**Supplementary Table 5 RNA sequence blasting between human and mouse RTEL1-derived circRNAs**

| **CircRNA ID** | **Spliced Length** | **Spliced Length of mmu-circ-0001109** | **Matched Bases** | **Matched percentage (≥157bp)** |
| --- | --- | --- | --- | --- |
| hsa_circ_0061166 | 1314 | 157 | 67 | 0.426751592 |
| hsa_circ_0061168 | 962 | 157 | 89 | 0.566878981 |
| hsa_circ_0092333 | 300 | 157 | 80 | 0.509554140 |

**Supplementary Table 6 RNA sequence blasting between human and mouse PRKAR2A-derived circRNAs**

| **CircRNA ID** | **Spliced Length** | **Spliced Length of mmu-circ-0001845** | **Matched Bases** | **Matched Percentage (≥434bp)** | **Matched percentage (<434bp)** |
| --- | --- | --- | --- | --- | --- |
| hsa_circ_0065501 | 4142 | 434 | 237 | 0.546082949 |  |
| hsa_circ_0124017 | 276 | 434 | 153 |  | 0.554347826 |
| hsa_circ_0124018 | 342 | 434 | 186 |  | 0.543859649 |
| hsa_circ_0065502 | 208 | 434 | 108 |  | 0.519230769 |
| hsa_circ_0124019 | 539 | 434 | 143 | 0.329493088 |  |
| hsa_circ_0065503 | 646 | 434 | 241 | 0.555299539 |  |
| hsa_circ_0124020 | 243 | 434 | 129 |  | 0.530864198 |
| hsa_circ_0124021 | 504 | 434 | 241 | 0.555299539 |  |
| hsa_circ_0124022 | 677 | 434 | 395 | 0.910138249 |  |
| hsa_circ_0124023 | 177 | 434 | 95 |  | 0.536723164 |
| hsa_circ_0124024 | 331 | 434 | 143 |  | 0.432024169 |
| hsa_circ_0124025 | 522 | 434 | 317 | 0.730414747 |  |
| hsa_circ_0124026 | 575 | 434 | 362 | 0.834101382 |  |
| hsa_circ_0124027 | 398 | 434 | 362 | 0.834101382 |  |
| hsa_circ_0124028 | 434 | 434 | 395 | 0.910138249 |  |
| hsa_circ_0124029 | 191 | 434 | 174 |  | 0.910994764 |
| hsa_circ_0065504 | 244 | 434 | 219 |  | 0.897540984 |
| hsa_circ_0065505 | 280 | 434 | 252 |  | 0.9 |
| hsa_circ_0124030 | 137 | 434 | 121 |  | 0.883211679 |
| hsa_circ_0065506 | 173 | 434 | 154 |  | 0.89017341 |
| hsa_circ_0124031 | 583 | 434 | 212 | 0.488479263 |  |

Supplementary Table 7 The correlations of 3 human PRKAR2A-derived circRNAs with the clinical characteristics of CAC patients

| **Variable** | **Hsa_circ_0124022** | | | **Hsa_circ_0124028** | | | **Hsa_circ_0124029** | | |
| --- | --- | --- | --- | --- | --- | --- | --- | --- | --- |
|  | **Low** | **High** | **P value** | **Low** | **High** | **P value** | **Low** | **High** | **P value** |
| Gender |  |  |  |  |  |  |  |  |  |
| Male | 22 | 25 | 0.621 | 27 | 20 | 0.137 | 21 | 26 | 0.322 |
| Female | 14 | 11 |  | 9 | 16 |  | 15 | 10 |  |
| Age at surgery |  |  |  |  |  |  |  |  |  |
| <mean age (48.23 years) | 16 | 21 | 0.346 | 12 | 25 | 0.004* | 20 | 17 | 0.638 |
| ≥mean age (48.23 years) | 20 | 15 |  | 24 | 11 |  | 16 | 19 |  |
| Smoking habit |  |  |  |  |  |  |  |  |  |
| Absent | 21 | 23 | 0.809 | 25 | 19 | 0.227 | 18 | 26 | 0.090 |
| Present | 15 | 13 |  | 11 | 17 |  | 18 | 10 |  |
| History of appendectomy |  |  |  |  |  |  |  |  |  |
| Absent | 31 | 34 | 0.429 | 35 | 30 | 0.107 | 32 | 33 | 1.000 |
| Present | 5 | 2 |  | 1 | 6 |  | 4 | 3 |  |
| UC extent |  |  |  |  |  |  |  |  |  |
| Pancolitis (E3) | 31 | 27 | 0.220 | 26 | 32 | 0.063 | 28 | 30 | 0.767 |
| Left side colitis (E2) | 4 | 9 |  | 10 | 3 |  | 7 | 6 |  |
| Proctitis (E1) | 1 | 0 |  | 0 | 1 |  | 1 | 0 |  |
| Preoperative therapy |  |  |  |  |  |  |  |  |  |
| Absent | 3 | 1 | 0.614 | 0 | 4 | 0.115 | 1 | 3 | 0.614 |
| Present | 33 | 35 |  | 36 | 32 |  | 35 | 33 |  |
| Type of surgery |  |  |  |  |  |  |  |  |  |
| Total colectomy | 33 | 34 | 1.000 | 35 | 32 | 0.357 | 36 | 31 | 0.054 |
| Other colon surgery | 3 | 2 |  | 1 | 4 |  | 0 | 5 |  |
| Disease duration (Period between UC onset and surgery, years) |  |  |  |  |  |  |  |  |  |
| <mean years (15.49) | 10 | 20 | 0.003* | 5 | 25 | 0.000* | 8 | 22 | 0.002* |
| ≥mean years (15.49) | 26 | 16 |  | 31 | 11 |  | 28 | 14 |  |
| Cancer stage |  |  |  |  |  |  |  |  |  |
| I/II | 15 | 8 | 0.129 | 18 | 5 | 0.002* | 16 | 7 | 0.042* |
| III/IV | 21 | 28 |  | 18 | 31 |  | 20 | 29 |  |
